# A potential space-making role in cell wall biogenesis for SltB1and DacB revealed by a beta-lactamase induction phenotype in *Pseudomonas aeruginosa*

**DOI:** 10.1128/mbio.01419-24

**Published:** 2024-06-26

**Authors:** Joël Gyger, Gabriel Torrens, Felipe Cava, Thomas G. Bernhardt, Coralie Fumeaux

**Affiliations:** 1 Institute of Microbiology, Lausanne University Hospital and University of Lausanne, Lausanne, Switzerland; 2 Laboratory for Molecular Infection Medicine Sweden (MIMS), Umeå Center for Microbial Research (UCMR), Umea, Sweden; 3 Department of Molecular Biology, Science for Life Laboratory (SciLifeLab), Umeå University, Umeå, Sweden; 4 Howard Hughes Medical Institute, Chevy Chase, Maryland, USA; 5 Department of Microbiology, Blavatnik Institute, Harvard Medical School, Boston, Massachusetts, USA; New York University School of Medicine, New York, New York, USA; Cornell University, Ithaca, New York, USA

**Keywords:** peptidoglycan, penicillin resistance, beta-lactamases, lytic transglycosylase

## Abstract

*Pseudomonas aeruginosa* encodes the beta-lactamase AmpC, which promotes resistance to beta-lactam antibiotics. Expression of *ampC* is induced by anhydro-muropeptides (AMPs) released from the peptidoglycan (PG) cell wall upon beta-lactam treatment. AmpC can also be induced via genetic inactivation of PG biogenesis factors such as the endopeptidase DacB that cleaves PG crosslinks. Mutants in *dacB* occur in beta-lactam-resistant clinical isolates of *P. aeruginosa*, but it has remained unclear why DacB inactivation promotes *ampC* induction. Similarly, the inactivation of lytic transglycosylase (LT) enzymes such as SltB1 that cut PG glycans has also been associated with *ampC* induction and beta-lactam resistance. Given that LT enzymes are capable of producing AMP products that serve as *ampC* inducers, this latter observation has been especially difficult to explain. Here, we show that *ampC* induction in *sltB1* or *dacB* mutants requires another LT enzyme called MltG. In *Escherichia coli*, MltG has been implicated in the degradation of nascent PG strands produced upon beta-lactam treatment. Accordingly, in *P. aeruginosa sltB1* and *dacB* mutants, we detected the MltG-dependent production of pentapeptide-containing AMP products that are signatures of nascent PG degradation. Our results therefore support a model in which SltB1 and DacB use their PG-cleaving activity to open space in the PG matrix for the insertion of new material. Thus, their inactivation mimics low-level beta-lactam treatment by reducing the efficiency of new PG insertion into the wall, causing the degradation of some nascent PG material by MltG to produce the *ampC*-inducing signal.

**IMPORTANCE:**

Inducible beta-lactamases like the ampC system of *Pseudomonas aeruginosa* are a common determinant of beta-lactam resistance among gram-negative bacteria. The regulation of *ampC* is elegantly tuned to detect defects in cell wall synthesis caused by beta-lactam drugs. Studies of mutations causing *ampC* induction in the absence of drug therefore promise to reveal new insights into the process of cell wall biogenesis in addition to aiding our understanding of how resistance to beta-lactam antibiotics arises in the clinic. In this study, the *ampC* induction phenotype for mutants lacking a glycan-cleaving enzyme or an enzyme that cuts cell wall crosslinks was used to uncover a potential role for these enzymes in making space in the wall matrix for the insertion of new material during cell growth.

## INTRODUCTION

*Pseudomonas aeruginosa* is an opportunistic pathogen that encodes multiple drug resistance mechanisms (1, 2). Infections with this bacterium can therefore be difficult to treat. One of its major resistance determinants is the AmpC beta-lactamase, which limits the effectiveness of many beta-lactam antibiotics against *P. aeruginosa* (3). Expression of the *ampC* gene is regulated in response to drug treatment. In the absence of antibiotic, it is expressed at low levels. However, treatment with some beta-lactams like cefoxitin, referred to as beta-lactamase inducers, results in potent induction of *ampC* expression and resistance. Other beta-lactams like piperacillin and ceftazidime (Caz) are not *ampC* inducers (4). These drugs therefore have anti-pseudomonal activity despite the ability of AmpC to hydrolyze them. Mutants with defects in *ampC* regulation causing constitutive beta-lactamase production are resistant to piperacillin and Caz. They are known to arise in the clinic and can result in treatment failures (5–9). There has thus been considerable interest in understanding *ampC* regulation and the mechanism by which mutations promote its aberrant overexpression.

**Fig 1:**
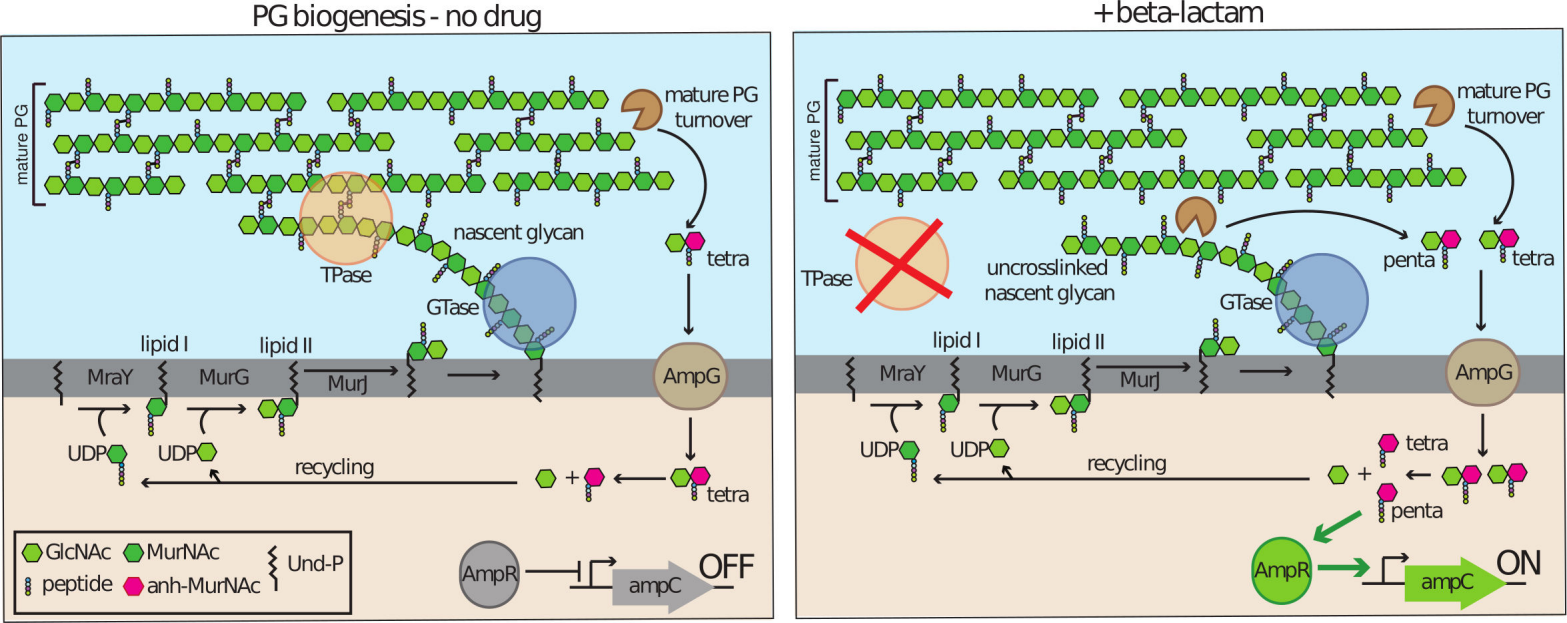
Overview of PG synthesis and *ampC* regulation. (Left) The PG matrix consists of glycan chains with the repeating unit of MurNAc and GlcNAc. Attached to the MurNAc sugars is a pentapeptide used to form crosslinks between adjacent glycans. PG synthesis starts in the cytoplasm, followed by the generation of lipid-linked precursors. Polymerization (GTase) and crosslinking (TPase) reactions at the membrane surface are used to form and insert nascent glycans into the mature matrix. The crosslinking reaction and carboxypeptidase enzymes act to rapidly convert the pentapeptide side chains to tetrapeptides via removal of the terminal D-Ala. The mature PG is subject to degradation by LT and EPs to generate anydro-MurNAc (anh-MurNAc) containing muropeptide (AMP) turnover products, which are imported to the cytoplasm by AmpG and recycled. The tetrapeptide AMP products, which are normally converted to tripeptide species upon import, are not thought to be good inducers for the activation of *ampC* expression by AmpR such that *ampC* is repressed in the absence of drug (16). (Right) Upon beta-lactam treatment, TPases are inhibited, and uncrosslinked nascent glycans are formed. These glycans are rapidly degraded generating pentapeptide AMP products that are also transported to the cytoplasm by AmpG (17, 18). These pentapeptide products are thought to be potent activators of AmpR for the induction of *ampC* expression (12, 13, 19, 20). Thus, *ampC* expression is a sensitive reporter for problems with PG crosslinking.

The expression level of *ampC* is linked to the status of peptidoglycan (PG) synthesis, and it responds to signals produced when beta-lactam antibiotics disrupt the process (3, 10–13). The PG cell wall surrounds most bacteria and is essential for maintaining cellular integrity. It is composed of glycan chains with a repeating disaccharide unit of N-acetylmuramic acid (MurNAc) and N-acetylglucosamine (GlcNAc). A pentapeptide that in most gram-negative bacteria has the sequence L-Ala-ɣ-D-Glu-meso-diaminopimelic acid (mDAP)-D-Ala-D-Ala is attached to the MurNAc sugar (14). It is used to form crosslinks between glycan chains, generating the matrix-like structure of the wall (Fig. 1) (15).

Two different types of synthases build the PG layer. The Class A penicillin-binding proteins (aPBPs) possess both glycosyltransferase (GTase) and transpeptidase (TPase) activities in a single polypeptide for the polymerization and crosslinking of PG, respectively. The other major synthases are composed of complexes between a SEDS family protein with GTase activity and a Class B penicillin-binding protein (bPBP) with TPase activity (21–23). Because the PG matrix is continuous, the insertion of new material requires the action of PG-cleaving enzymes to make space for the incoming nascent glycans (15). Currently, endopeptidases (EPs) that cut the peptide crosslinks are thought to be the main PG-processing enzymes that function as space makers (15, 24–27).

Beta-lactams covalently modify the TPase active sites of PBPs and inhibit PG crosslinking (28). These drugs do not block the GTase activity of the polymerase enzymes. Thus, uncrosslinked PG glycans are produced following drug treatment (29). In the related gram-negative bacterium *Escherichia coli*, these uncrosslinked strands have been shown to be rapidly degraded by lytic transglycosylase (LT) enzymes (29, 30). LTs cleave the glycan strand and generate disaccharide–peptide products with a 1,6-anhydro linkage on the MurNAc sugar (31). These so-called anhydro-muropeptides (AMPs) are produced by LTs during normal growth as these enzymes help promote the high turnover of the mature PG observed per generation (ca. 40%/generation) (Fig. 1) (32). In this case, the turnover products are primarily in the tetrapeptide form (33) because the terminal D-Ala of the stem peptide is either removed in the process of crosslinking or rapidly trimmed by enzymes called D-Ala-D-Ala carboxypeptidases. However, when nascent PG is processed by LTs during beta-lactam treatment, the AMPs produced are in the pentapeptide form (29). These AMPs are likely to be the preferred inducer of the AmpR regulator, converting it to an activator of *ampC* expression (Fig. 1) (12, 13, 19, 20). Thus, *ampC* regulation is elegantly tuned to detect problems with nascent PG crosslinking as a proxy for the presence of beta-lactams.

Genetic inactivation of DacB, a PG-processing enzyme with both PG EP and carboxypeptidase activity (34), has been known to induce *ampC* expression and promote beta-lactam resistance for some time (7–9). However, the mechanism by which the *ampC*-inducing signal is generated in *dacB* mutants has remained unclear. Mutations that inactivate LT enzymes have also been found to increase the expression of beta-lactamase genes in several different gram-negative organisms (35–38). Given that LTs are typically associated with the production of the *ampC*-inducing signal, these results have been difficult to explain.

In this report, we used a *P. aeruginosa* strain with a *lacZ* reporter gene fused to the *ampC* promoter (P*_ampC_::lacZ*) (39) to find that defects in the LT enzyme SltB1 aberrantly induce *ampC*. In an effort to uncover the mechanism that leads to *ampC* induction in *sltB1* mutants, we found that the activation of *ampC* expression in cells lacking SltB1 or DacB requires MltG, an LT enzyme previously implicated in the turnover of nascent PG in *E. coli* and the induction of *ampC* following beta-lactam treatment in *P. aeruginosa* (30, 40, 41). Accordingly, in *sltB1* and *dacB* mutants, we detected the MltG-dependent production of pentapeptide-containing AMP products that are signatures of nascent PG degradation. Our results therefore support a model in which SltB1 and DacB use their PG-cleaving activity to open space in the PG matrix for the insertion of new material. Thus, in the absence of these enzymes, the efficiency of incorporation of new PG strands into the wall is reduced, mimicking low-level beta-lactam treatment to cause a fraction of nascent PG material to be degraded by MltG to produce the *ampC*-inducing signal.

## RESULTS

### Inactivation of *sltB1* induces *ampC* expression and promotes beta-lactam resistance

**Fig 2:**
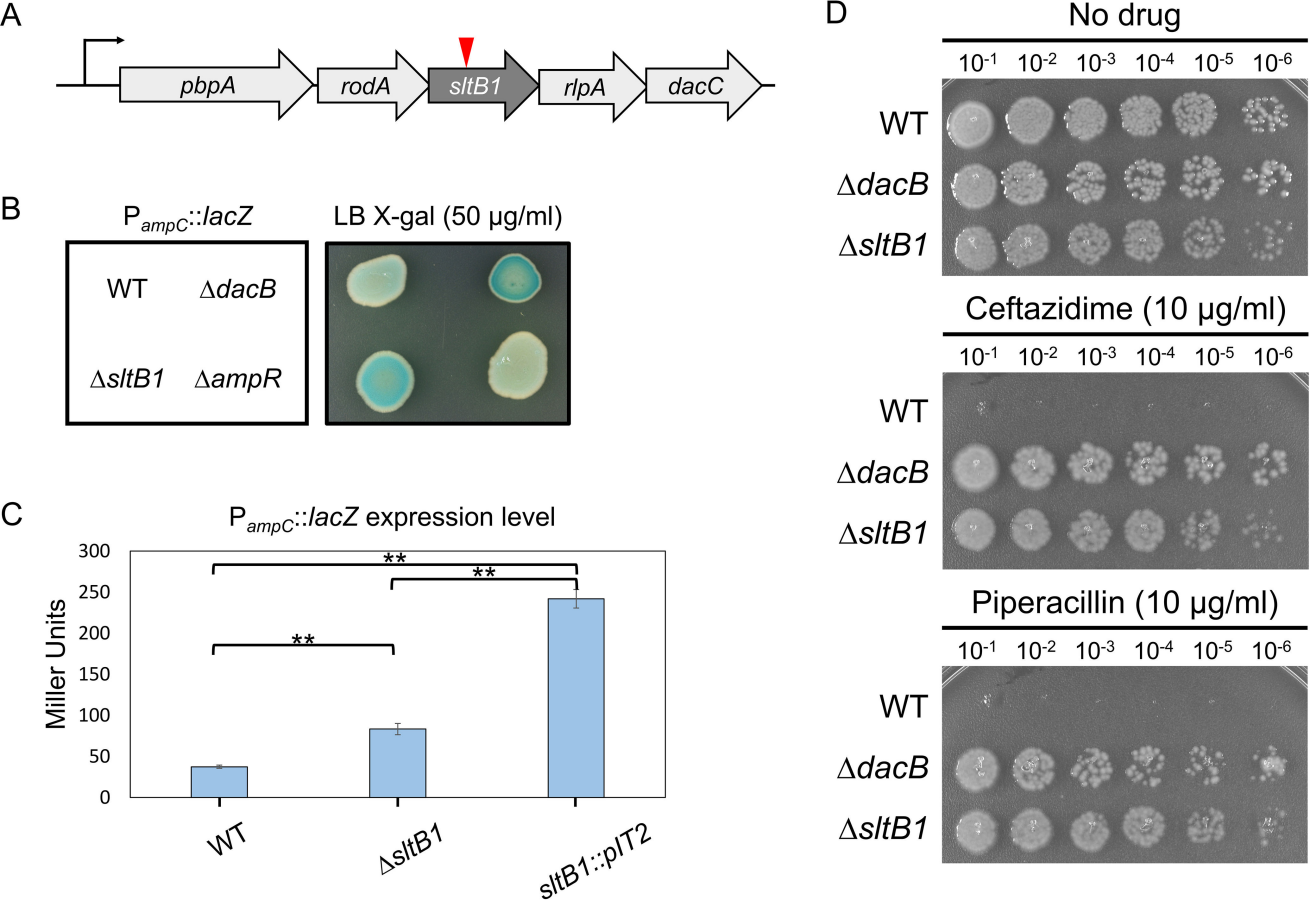
Inactivation of SltB1 promotes P*_ampC_::lacZ* induction and beta-lactam resistance. (**A**) Diagram of the genetic locus harboring *sltB1*. The *pbpA* and *rodA* genes encode the RodA-PBP2 complex that forms the essential PG synthase of the cell elongation system (15). The *rlpA* gene encodes an LT enzyme that functions in cell division by promoting daughter cell separation (44), and *dacC* encodes a carboxypeptidase that trims PG peptides (45). (**B**) Culture aliquots (5 µL) of strains CF262 (PAO1 WT), CF268 (∆*dacB*), CF1143 (∆*sltB1*), and CF604 (∆*ampR*) containing the P*_ampC_::lacZ* reporter were spotted onto lysogeny broth (LB) agar containing X-gal (50 µg/mL), grown overnight at 30°C, and photographed. (**C**) Beta-galactosidase activity in Miller units was measured in liquid cultures of the indicated strains. Results shown are the average of three assays with two or three biological replicates per strain, except for the *sltB1*::Tn strain (no biological replicate), and the error bars represent the standard deviation. A one-way analysis of variance revealed that there was a statistically significant difference in *ampC* expression level between at least two strains [*F*(106610.925, 6211.075) = 188.81114, *P* < 0.00001]. Tukey’s honestly significant difference test for multiple comparisons found that the mean value of *ampC* expression was significantly different between WT and ∆*sltB1*, WT and *sltB1*::Tn, and ∆*sltB1* and *sltB1*::Tn (*P* = 0.01). (**D**) Cultures of strains PAO1 (WT), CF155 (∆*dacB*), and CF1105 (∆*sltB1*) were serially diluted, and 5 µL of each dilution was spotted onto LB agar supplemented with Caz (10 µg/mL) or piperacillin (10 µg/mL) as indicated.

A wild-type (WT) *P. aeruginosa* strain (PAO1) carrying a P*_ampC_::lacZ* reporter (39) inserted at the *attB* locus was mutagenized with a transposon. The resulting mutant library was plated on agar containing X-gal to identify insertions causing *ampC* expression in the absence of beta-lactam treatment as a mean to identify factors required for proper PG biogenesis. Prior screens using this library identified transposon mutants in the *mupP* gene, which led to its functional characterization as an enzyme important for the recycling of PG fragments (42). We continued to screen the library and identified an additional mutant forming solid blue colonies on X-gal agar, a phenotype that is indicative of aberrant *ampC* induction. PCR-based mapping revealed that this isolate had a transposon inserted in the *sltB1* (*PA4001*) gene (Fig. 2A), encoding the LT enzyme SltB1 that is related to *E. coli* MltB (43).

A deletion mutation of *sltB1* was previously found to promote beta-lactam resistance (35, 36). However, in these studies, the authors did not detect elevated AmpC production in an *sltB1* mutant by nitrocefin hydrolysis assays or immunoblotting despite observing an *ampC* requirement for the resistance phenotype and an increased AmpC level when the *sltB1* mutation was combined with deletion of *dacB* (35, 36). It was therefore concluded that beta-lactam resistance upon SltB1 inactivation was not due to *ampC* induction but instead was likely to result from the inactivation of a lysis pathway involving cell wall damage caused by SltB1 (35, 36). The identification of the transposon insertion in *sltB1* in our screen argues against this interpretation and for a more direct role of SltB1 inactivation in *ampC* induction.

To validate the results from the screen, an in-frame deletion of *sltB1* similar to the previously published deletion (35, 36) was generated in the reporter strain. An aliquot of the mutant culture was spotted on agar containing X-gal alongside cultures of WT, a ∆*dacB* mutant known to promote high-level *ampC* induction, and a mutant lacking *ampR* that is defective for *ampC* expression (8, 46). As expected, the spots from the WT and ∆*ampR* culture remained white, whereas that from the ∆*dacB* culture turned blue (Fig. 2B). The spot from the ∆*sltB1* culture also turned blue, indicating that SltB1 inactivation induces the *ampC* promoter in the absence of beta-lactams (Fig. 2B). Quantification of beta-galactosidase activity confirmed that the P*_ampC_::lacZ* reporter was induced in the ∆*sltB1* mutant and the original *sltB1*::Tn isolate relative to WT (Fig. 2C). Notably, the transposon insertion allele led to a greater induction of the reporter than the deletion, which is potentially due to effects of the insertion on the expression of nearby genes encoding other PG biogenesis proteins (Fig. 2A). We conclude that inactivation of SltB1 induces expression from the *ampC* promoter.

**Fig 3:**
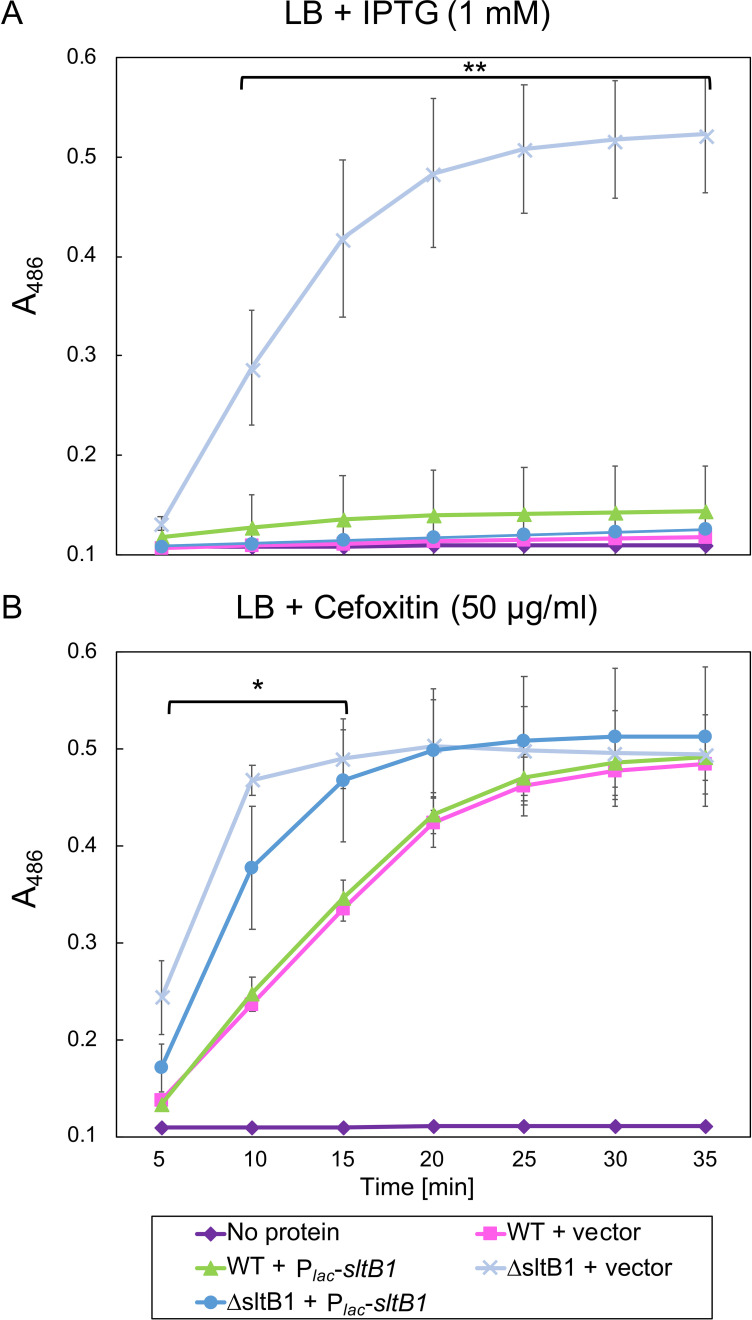
AmpC activity is elevated upon inactivation of SltB1. Assays of nitrocefin hydrolysis by cell lysates of PAO1 (WT) or CF1105 (∆*sltB1*) with plasmids pKHT103 (vector control) or pCF533 (P*_lac_::sltB1*) as indicated. Data are the mean of three independent assays each for two biological replicates with the error bars indicating the standard error. (**A**) Cells were grown in LB with IPTG (1 mM). A one-way analysis of variance revealed that there was a statistically significant difference in nitrocefin hydrolysis level between at least two strains [*F*(3, 25) = 72.23, *P* < 0.00001]. Tukey’s honestly significant difference test for multiple comparisons found that the mean value of nitrocefin hydrolysis level was significantly different between the ∆*sltB1* + vector and all the other strains grown in presence of IPTG (WT + vector, WT + *sltB1*, and ∆*sltB1 + sltB1*), as indicated by the asterisks (*P* = 0.01). (**B**) Cells were grown in presence of the *ampC* inducer cefoxitin (50 µg/mL). A one-way analysis of variance revealed that there was a statistically significant difference in nitrocefin hydrolysis level between at least two strains [*F*(3, 33) = 10.66, *P* < 0.000076]. Tukey’s honestly significant difference test for multiple comparisons found that the mean value of nitrocefin hydrolysis level was significantly different between the ∆*sltB1* (+vector or +*sltB1*) and WT (+vector or +*sltB1*) strains for the early time points, as indicated by the asterisk (*P* = 0.05).

To monitor the effect of SltB1 inactivation on native P*_ampC_* induction, we tested the beta-lactam resistance of mutant cells and measured the production of AmpC using a nitrocefin hydrolysis assay. Consistent with prior results, the ∆*sltB1* strain was resistant to the antipseudomonal beta-lactams Caz and piperacillin, as was a control ∆*dacB* strain (Fig. 2D). Similarly, and in contrast to previous work (35), a ∆*sltB1* mutant with an empty vector showed elevated AmpC activity in the nitrocefin assay relative to WT cells (Fig. 3A). Deletion of *sltB1* did not strongly affect the induction of AmpC activity by the beta-lactam cefoxitin (Fig. 3B). The *sltB1* gene is in a putative operon that includes several genes encoding PG synthesis and remodeling proteins, including the downstream *rlpA* and *dacC* genes that encode an LT enzyme and a D-Ala-D-Ala carboxypeptidase, respectively (Fig. 2A) (44, 45). Notably, normal beta-lactam sensitivity and AmpC activity were restored to ∆*sltB1* cells upon expression of *sltB1* from a plasmid (Fig. S1), indicating that the phenotype of the deletion allele was caused by the inactivation of SltB1 and not an effect of the deletion on the expression of the nearby genes encoding PG biogenesis factors. Expression of a catalytic mutant of SltB1, SltB1(E135A), failed to complement the beta-lactam resistance phenotype of the *sltB1* deletion (Fig. S1), indicating that it is the loss of SltB1 activity that results in *ampC* induction.

### AmpC production in ∆*sltB1* cells is induced via the canonical mechanism

**Fig 4:**
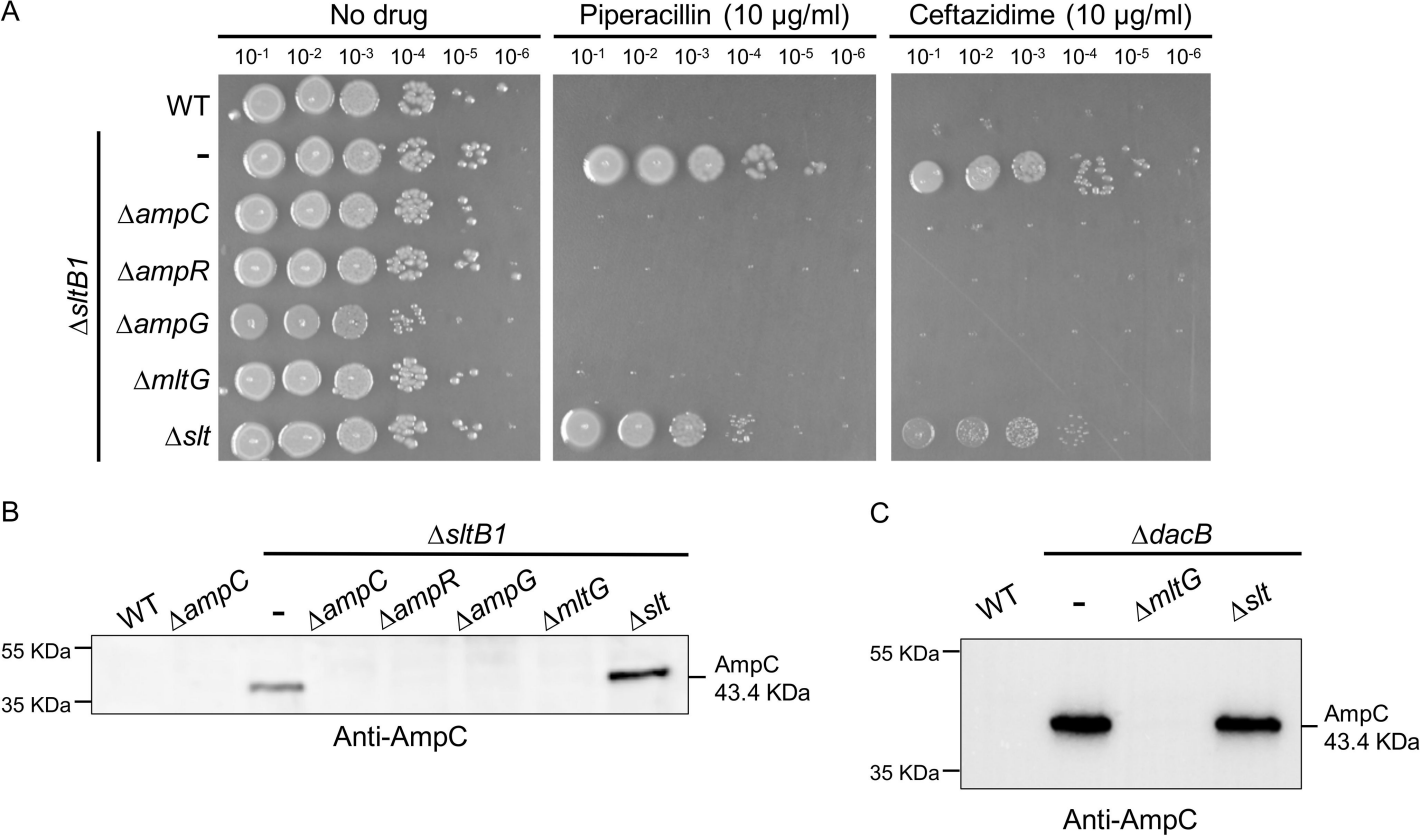
Requirements for beta-lactam resistance and AmpC induction in ∆*sltB1* and ∆*dacB* cells. (**A**) Cultures of strains PAO1 (WT), CF1105 (∆*sltB1*), CF368 (∆*sltB1* ∆*ampC*), CF370 (∆*sltB1* ∆*ampR*), CF372 (∆*sltB1* ∆*ampG),* CF1416 (∆*sltB1* ∆*mltG*), and CF378 (∆*sltB1* ∆*slt*) were serially diluted, and 5 µL of each dilution was spotted onto LB agar with or without drug (10 µg/mL) as indicated. (**B**) Immunoblot for AmpC protein using the strains from panel **A**. (**C**) Immunoblot for AmpC protein using the strains PAO1 (WT), CF155 (∆*dacB*), CF256 (∆*dacB* ∆*mltG*), and CF1446 (∆*dacB* ∆*slt*).

The results presented thus far suggest that mutants lacking SltB1 are resistant to beta-lactam treatment via the induction of *ampC* expression as opposed to alternative mechanisms proposed previously (35, 36). To further test this possibility, we investigated the requirements for resistance in the ∆*sltB1* background. Deletion of *ampC* or the *ampR* gene encoding the *ampC* transcriptional activator eliminated the beta-lactam resistance phenotype of ∆*sltB1* cells and resulted in the loss of AmpC accumulation as determined by immunoblot (Fig. 4A and B). Beta-lactam resistance and AmpC accumulation were also blocked in the ∆*sltB1* mutant by inactivation of AmpG, the transporter that imports the AMP products from cell wall degradation that are sensed by AmpR to activate *ampC* expression (Fig. 1, 4A and B) (17, 18). We therefore conclude that *ampC* is being induced in the ∆*sltB1* cells by the canonical induction mechanism.

### MltG is required for *ampC* induction in ∆*sltB1* and ∆*dacB* cells

**Fig 5:**
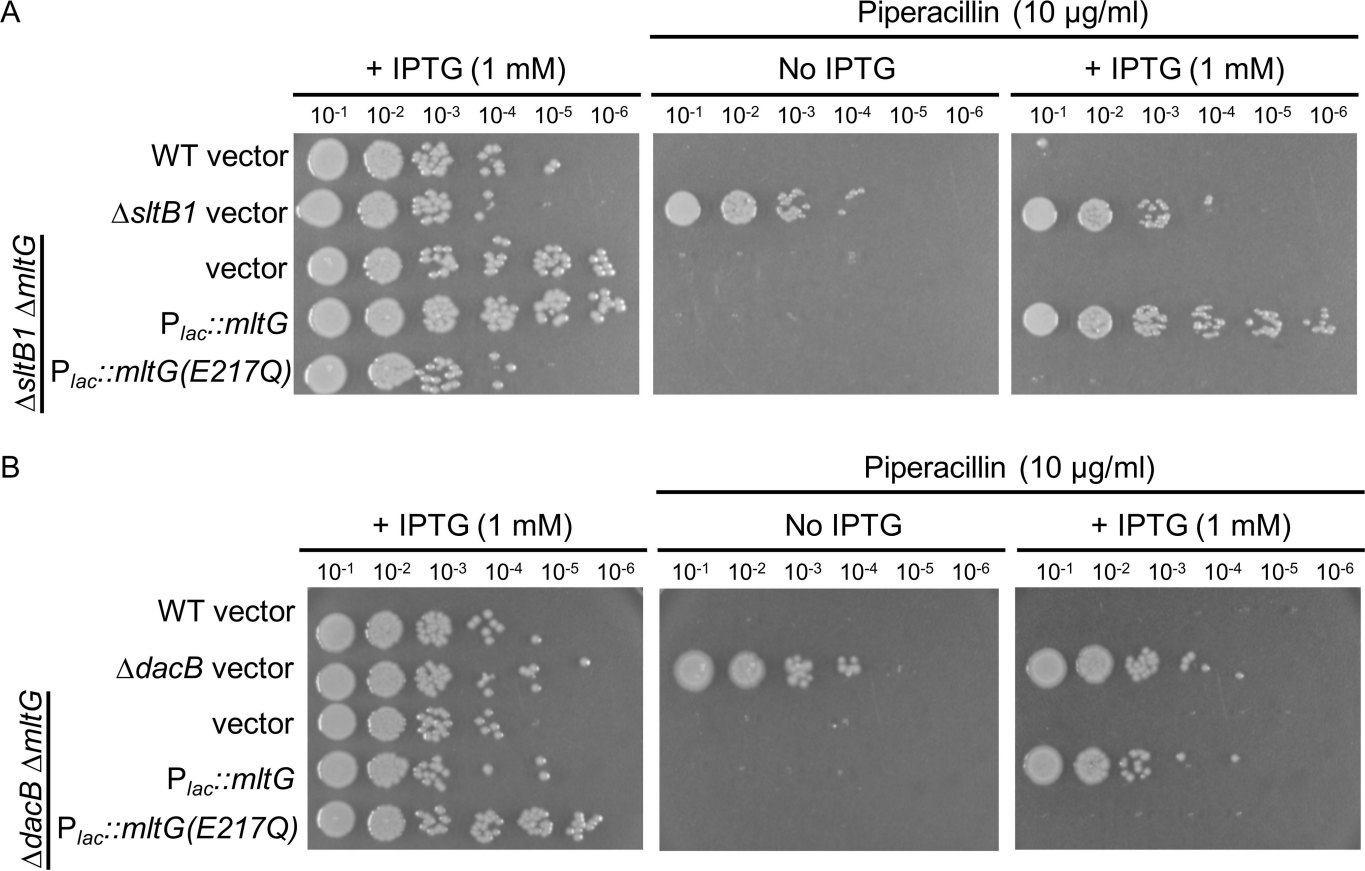
MltG is required for ampC induction in cells defective for SltB1 or DacB. (**A**) Cultures of strains PAO1 (WT), CF1105 (∆*sltB1*), and CF1416 (∆*sltB1* ∆*mltG*) containing plasmids pKHT103 (vector control), pCF658 (P*_lac_::mltG*), or pCF1328 [P*_lac_::mltG(E217Q*)] were serially diluted, and 5 µL of each dilution was spotted onto LB agar with or without piperacillin (10 µg/mL) and/or IPTG (1 mM) as indicated. (**B**) Cultures of strains PAO1 (WT), CF155 (∆*dacB*), and CF256 (∆*dacB* ∆*mltG*) containing plasmids pKHT103 (vector control), pCF658 (P*_lac_::mltG*), or pCF1328 [P*_lac_::mltG(E217Q*)] were serially diluted, and 5 µL of each dilution was spotted onto LB agar with or without piperacillin (10 µg/mL) and/or IPTG (1 mM) as indicated.

In *E. coli*, uncrosslinked PG strands are generated upon beta-lactam treatment, and these strands are rapidly degraded by the LT enzymes Slt and MltG, with Slt playing the predominant role (29, 30). The action of these LTs generates pentapeptide-containing AMP products that in *P. aeruginosa* would serve as inducers of *ampC* expression. We therefore investigated whether Slt or MltG is required for *ampC* induction in ∆*sltB1*. Deletion of *slt* did not have a strong effect on the piperacillin resistance of ∆*sltB1* cells and actually appeared to increase AmpC production based on immunoblotting (Fig. 4A and B). By contrast, inactivation of MltG restored piperacillin and Caz sensitivity to the ∆*sltB1* mutant and resulted in AmpC being undetectable in these cells (Fig. 4A and B). We also tested the requirement for Slt or MltG for *ampC* induction in ∆*dacB* cells. As with ∆*sltB1* cells, AmpC protein accumulation was abolished by MltG inactivation but not the loss of Slt function (Fig. 4C). Additionally, AmpC activity in ∆*dacB* cells as assessed by nitrocefin hydrolysis was also found to be dependent on MltG but not Slt (Fig. S2). Notably, the piperacillin and Caz sensitivity of ∆*sltB1* ∆*mltG* and ∆*dacB* ∆*mltG* cells was converted back to resistance upon expression of *mltG*(WT) from a plasmid but not by *mltG*(E217Q) encoding a catalytically dead MltG enzyme (Fig. 5; Fig. S3). Similarly, the induction of the P*_ampC_::lacZ* reporter in ∆*sltB1* ∆*mltG* and ∆*dacB* ∆*mltG* cells was restored by plasmid-based production of MltG(WT) but not MltG(E217Q) (Fig. S3). Thus, mutants lacking SltB1 or DacB similarly require MltG activity for *ampC* induction and beta-lactam resistance.

### MltG-dependent accumulation of pentapeptide-containing AMPs in ∆*sltB1* and ∆*dacB* cells

**Fig 6:**
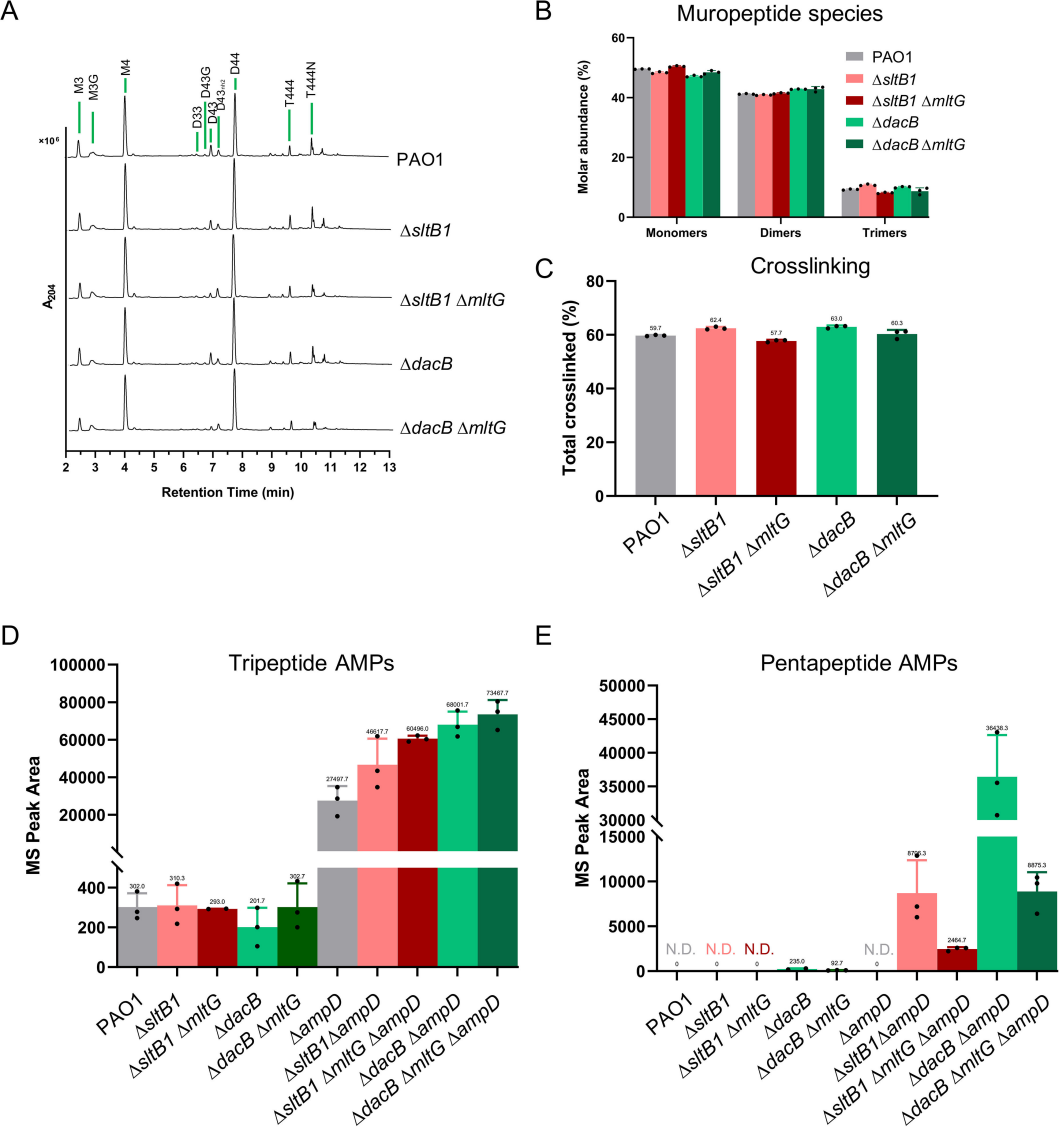
Accumulation of pentapeptide AMPs in ∆*sltB1* and ∆*dacB* mutants confirmed by soluble muropeptides analyses. (**A**) UPLC PG analysis, with characteristic peaks labeled as follows: M, monomeric muropeptide (uncrosslinked); D, dimeric muropeptide (crosslink connecting two muropeptides); T, trimeric muropeptide (crosslinks connecting three muropeptides). Numbers refer to the status of the peptide side chain (3 = tripeptide, 4 = tetrapeptide). Strains used for analysis were: PAO1 (WT), CF1105 (∆*sltB1*), CF1416 (∆*sltB1* ∆*mltG*), CF155 (∆*dacB*) and CF256 (∆*dacB* ∆*mltG*). (**B**) Abundance of muropeptide species (monomers, dimers, and trimers) in the same strains as in panel **A**. (**C**) Analyses of PG crosslinking in the same strains as in panel **A**. (**D** and **E**) Quantification of tripeptide-AMPs (**D**) or pentapeptide AMPs (**E**) in strains PAO1 (WT), CF1105 (∆*sltB1*), CF1416 (∆*sltB1* ∆*mltG*), CF155 (∆*dacB*), CF256 (∆*dacB* ∆*mltG*), CF186 (∆*ampD*), CF1585 (∆*sltB1* ∆*ampD*), CF1591 (∆*sltB1* ∆*mltG* ∆*ampD*), CF189 (∆*dacB* ∆*ampD*), and CF1588 (∆*dacB* ∆*mltG* ∆*ampD*). N.D.: non-detected.

The results thus far are consistent with the production of uncrosslinked PG strands in ∆*sltB1* and ∆*dacB* mutants that are turned over by MltG to produce the AMP products that induce *ampC* expression. To investigate this possibility, we first performed muropeptide analysis to determine whether the overall structure of PG was altered in cells lacking SltB1 or DacB. No major changes in PG composition were detected whether or not the ∆*sltB1* or ∆*dacB* mutant cells possessed functional MltG (Fig. 6A through C). Thus, the bulk of PG synthesis appears to be proceeding normally in cells lacking SltB1 or DacB as expected based on the robust growth of the mutants. However, based on their *ampC* induction phenotypes, we suspected that in cells inactivated for DacB or SltB1, a subset of PG synthetic complexes may encounter problems with PG crosslinking such that uncrosslinked material is generated and rapidly degraded to produce pentapeptide-containing AMP products. We therefore measured the levels of AMP products produced in WT and mutant cells. In cells with a functional PG recycling system, equivalent levels of tripeptide-containing AMPs were detected in all strains (Fig. 6D). Pentapeptide-containing AMPs were undetectable presumably due to their low steady-state levels in cells capable of recycling the AMPs (Fig. 6E). We therefore transitioned to monitoring AMPs in cells defective for the AmpD recycling amidase (Fig. 1), which would prevent AMP recycling and raise their steady-state levels to improve detection of the pentapeptide-containing species. Cells lacking AmpD showed an elevated level of tripeptide-containing AMPs as expected, but there were no major increases in the levels of these species detected in ∆*ampD* cells inactivated for SltB1 or DacB whether or not they had an additional defect in MltG (Fig. 6D). However, in the ∆*ampD* background, cells defective for SltB1 or DacB showed a significant increase in the level of pentapeptide-containing AMPs that was MltG-dependent (Fig. 6E). This result indicates that there is an elevated level of nascent PG degradation in cells inactivated for SltB1 or DacB and that this degradation is most likely performed by MltG. We therefore infer that SltB1 and DacB are functioning as space-making enzymes to promote the efficient incorporation of new PG strands into the matrix for its expansion (see Discussion).

### Overexpression of catalytically inactivated SltB1 induces *ampC* expression

**Fig 7:**
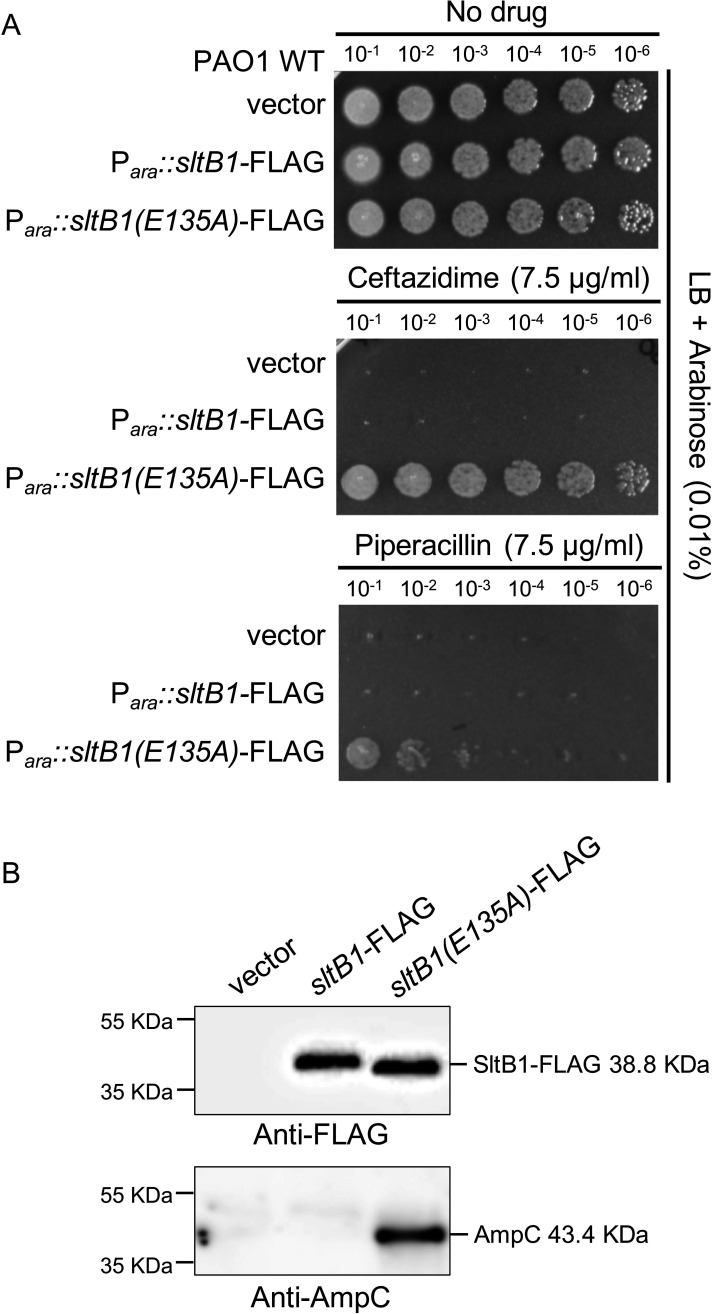
Overproduction of catalytically inactive SltB1 induces AmpC production and promotes beta-lactam resistance. (**A**) Cultures of strain PAO1 (WT) with plasmids pJN105 (vector control), pCF1009 (P*_ara_::sltB1-FLAG*), or pCF1010 [P*_ara_::sltB1(E135A)-FLAG*] were serially diluted, and 5 µL of each dilution was spotted onto LB agar supplemented with arabinose (0.01%) and Caz (7.5 µg/mL) or piperacillin (7.5 µg/mL) as indicated. (**B**) Immunoblots for FLAG-tagged proteins and AmpC protein using the strains from panel **A**. The cause of the shift in molecular weight for SltB1(E135A)-FLAG is not known.

PG-cleaving enzymes have been proposed to function as part of multi-protein complexes that help coordinate their activities with those of PG synthases (14). Notably, SltB1 has been found to interact with PBP2 (43, 47), a bPBP that is involved in cell elongation and shape determination (23). We therefore wondered whether SltB1 might have a limited number of binding sites within the PG layer and/or with protein-binding partners in the cell, which are required for its function. To test this possibility, we overproduced a FLAG-tagged variant of either SltB1(WT) or the catalytically inactive variant SltB1(E135A) in WT cells and monitored beta-lactam resistance and AmpC production. Overexpression of *sltB1(WT)-FLAG* did not alter the Caz or piperacillin sensitivity of the WT strain, nor did it lead to the detectable induction of AmpC production (Fig. 7). However, overproduction of SltB1(E135A)-FLAG to levels equivalent to that of the WT protein conferred a Caz and piperacillin resistance phenotype to otherwise WT cells and led to AmpC production (Fig. 7). This resistance phenotype required *ampC*, *ampR*, and *mltG* similar to that of the *sltB1* deletion (Fig. S4). Thus, overproduction of SltB1(E135A)-FLAG is likely to saturate a critical protein partner or PG binding site used by SltB1(WT) to perform its function, leading to an SltB1-defective phenotype. The binding partner may be a PG synthase like PBP2, and SltB1 may work with this and other PG synthases to promote the efficient incorporation of nascent PG strands into the matrix for PG expansion. Accordingly, when *ampC* is deleted to remove its influence on beta-lactam resistance, cells lacking SltB1 display increased sensitivity to a range of different beta-lactams but not the aminoglycoside tobramycin or the outer membrane-impermeable PG synthesis inhibitor vancomycin (Table 1). Notably, SltB1 inactivation also resulted in a mild increase in sensitivity to the MreB antagonist A22 that disrupts the function of the PBP2-containing cell elongation system (Table 1) (48). We therefore conclude that SltB1 is likely to participate in cell wall synthesis and expansion, potentially as a component of a PG synthesis complex, a role not typically associated with LT enzymes capable of cleaving PG strands.

**TABLE 1:** MIC of selected antibiotics

Antibiotics	∆*ampC*	∆*ampC* ∆*sltB1*
A22^*a*^	3	0.75–1.5
Aztreonam^*b*^	2–4	0.75–2
Carbenicillin^*a*^	50–100	25–50
Cefepime^*b*^	1–1.5	0.25–0.75
Cefoxitin^*a*^	100	25–50
Ceftazidime^*b*^	1–1.5	0.38–0.5
Mecillinam^*a*^	150	37.5–75
Meropenem^*b*^	0.19–0.25	0.125–0.19
Piperacillin^*a*^	5	2.5
Tobramycin^*a*^	1	1
Vancomycin^*b*^	>256	>256

^*a*^ MIC determined by broth microdilution.

^*b*^ MIC determined by *E* test strip.

## DISCUSSION

Mutations that inactivate LT enzymes have been found to increase the expression of beta-lactamase genes in several different gram-negative organisms (35–38). However, the mechanism behind this phenomenon and how it relates to the role(s) of LTs in cell wall biogenesis has remained unclear. It was originally reported that mutants defective for SltB1 in *P. aeruginosa* confer elevated beta-lactam resistance via the inactivation of a cell death pathway involving autolysis by this PG-cleaving enzyme (35, 36). This mechanism was proposed because the authors did not detect elevated AmpC activity or protein levels in extracts from ∆*sltB1* cells despite the resistance phenotype being dependent on *ampC* and the detection of elevated AmpC in ∆*dacB* cells inactivated for SltB1 (35, 36). By contrast, our results indicate that ∆*sltB1* cells are induced for *ampC* expression, which was shown in several ways: (i) via a P*_ampC_::lacZ* reporter gene, (ii) AmpC activity assays, and (iii) immunoblotting for AmpC. Furthermore, induction was shown to depend on the importer AmpG that transports AMP products into the cytoplasm and the AmpR activator that stimulates *ampC* expression when it senses these molecules (17, 18, 46). Why the previous reports did not also observe *ampC* induction in their *sltB1* mutant is not known (35, 36). Nevertheless, the results presented here strongly support the conclusion that SltB1 inactivation confers beta-lactam resistance by activating *ampC* expression via the canonical mechanism involving the sensing of AMP turnover products.

Inactivation of DacB has also been associated with *ampC* induction through the canonical sensing of AMP turnover products (7–9). Pentapeptide-containing AMP molecules were previously shown to be produced in ∆*dacB* cells, providing evidence that the pentapeptide species is the most potent inducing molecule for the activation of *ampC* expression (49). DacB is known to have both PG carboxypeptidase and PG EP activity *in vitro* (34). However, cells lacking DacB were not found to have a significant increase in pentapeptides in their cell wall, indicating that it is not a major carboxypeptidase *in vivo* (34, 49). Thus, the pentapeptide-containing AMPs detected in ∆*dacB* cells are not generated via turnover of the mature PG sacculus. How they are produced has remained unclear.

An important clue to the mechanism by which DacB or SltB1 inactivation causes *ampC* induction came from the observation that it requires the LT enzyme called MltG. In *E. coli*, this inner membrane-anchored LT has been implicated in the turnover of nascent PG glycans following cefsulodin treatment of *E. coli* (30). Additionally, LT activity has been shown to degrade uncrosslinked PG strands in the periplasm of other gram-negative bacteria (50). In *P. aeruginosa*, MltG was found to be a key target of bulgecin A, an LT inhibitor that sensitizes *P. aeruginosa* and other gram-negative bacteria to beta-lactams (41). The implication of this finding is that MltG is likely involved in the turnover of uncrosslinked strands produced upon beta-lactam treatment, preventing the toxic side effects of these glycans (29) and providing the pentapeptide-containing AMP products for *ampC* induction. Accordingly, we showed that pentapeptide-containing AMPs are produced in ∆*dacB* and ∆*sltB1* cells and that their production is MltG-dependent. Notably, MltG was also recently shown to be required for *ampC* induction and beta-lactam resistance in a clinical isolate of *P. aeruginosa* (40). Therefore, based on the MltG requirement for *ampC* activation in ∆*sltB1* and ∆*dacB* cells, we infer that the inactivation of these enzymes causes problems with the incorporation of nascent PG strands into the cell wall matrix such that uncrosslinked nascent PG strands are produced and rapidly degraded by MltG to generate the AMP signals that induce beta-lactamase expression. Given that ∆*sltB1* and ∆*dacB* mutants grow normally and do not show a decrease in overall PG crosslinks in their mature sacculi, the defect in nascent PG incorporation in these cells is likely to be relatively minor. It is only through the high sensitivity of the *ampC* regulatory system for pentapeptide-containing AMPs that the roles for these enzymes in nascent PG incorporation are revealed.

How might DacB and SltB1 be promoting the insertion of nascent PG strands into the mature wall? The simplest explanation is that they are functioning as space-making enzymes that cleave linkages in the cell wall to make room for the insertion of new material to expand the PG matrix during growth. PG EPs have previously been implicated as space makers (15, 24–27). DacB is therefore likely to similarly use its PG EP activity to provide sites for nascent PG incorporation. Unlike EPs, LTs have not traditionally been thought to function in a space-making capacity, but a recent report suggests the LT enzyme MltD in *E. coli* performs such a role (51). Based on the *ampC* induction phenotype upon SltB1 inactivation in *P. aeruginosa*, we propose that this LT enzyme also functions as a space maker.

A major outstanding question in the field is how the activity of space-making enzymes is coordinated with PG synthesis to prevent imbalanced PG cleavage and cell lysis. Notably, DacB has been shown to interact with the aPBP-type PG synthase PBP1a in *E. coli* (52), raising the possibility that it may work in complex with PBP1a to promote the insertion of new material made by this enzyme. SltB1, on the other hand, has been found to interact with PBP2 in *P. aeruginosa* (43, 47). PBP2 is a bPBP with TPase activity that together with the SEDS GTase RodA forms the essential cell wall synthase of the Rod system (elongasome) that is responsible for cell elongation and shape determination (15, 21–23). Although the physiological relevance of the SltB1-PBP2 interaction has yet to be demonstrated, the location of the *sltB1* gene just downstream of the genes encoding RodA and PBP2 (Fig. 2A) suggests that SltB1 may be a non-essential component of the Rod system that cleaves glycan strands in the mature PG matrix that may interfere with the insertion of new PG material during cell wall expansion. Consistent with this model, catalytically inactive SltB1 exerts a dominant-negative, ∆*sltB1-*like phenotype indicating that SltB1 is likely to have a limited number of protein partner binding sites in the cell, which are required for its function. Cells lacking SltB1 show increased sensitivity to the Rod system antagonist A22 (Table 1) (48), further supporting an auxiliary role for SltB1 in the function of the cell elongation system.

In conclusion, we have used an *ampC* induction phenotype in *P. aeruginosa* to reveal a potential function for the DacB EP and the SltB1 LT enzyme as space makers promoting the insertion of new PG strands into the mature wall. Such a function is only commonly ascribed to EPs that cut PG crosslinks (15, 24–27). However, the PG matrix is unlikely to be so neatly arranged that crosslinks are the only impediment to the insertion of nascent glycans. Active synthases are also likely to encounter mature glycans that cross their path and require removal for the new strand to be effectively incorporated. Thus, based on our findings for SltB1 and recent work on MltD in *E. coli* (51), it is likely that a subset of the many LTs encoded by gram-negative bacteria function in a space-making capacity similar to that of the EPs.

## MATERIALS AND METHODS

### Media, bacterial strains, and plasmids

*P. aeruginosa* PAO1 cells were grown in lysogeny broth (LB) (1% tryptone, 0.5% yeast extract, and 0.5% NaCl). As indicated, the medium was supplemented with 1-mM isopropyl β-D-1-thiogalactopyranoside (IPTG), 0.01% arabinose, 5% sucrose, or 50-µg/mL X-gal (5-bromo-4-chloro-3-indolyl-beta-D-galactopyranoside). For plasmid maintenance or integration, gentamicin (Gm), tetracycline (Tet), and carbenicillin (Carb) were used at a concentration of 30, 50, and 200 µg/mL, respectively. For AmpC beta-lactamase induction, cefoxitin (Fox) was used at a concentration of 50 µg/mL. Unless otherwise indicated, anti-pseudomonal antibiotics for viability/sensitivity assays were used at 10 µg/ml (piperacillin; Pip or Caz). All *P. aeruginosa* strains used in the reported experiments are derivatives of PAO1. *E. coli* cells were grown in LB. For plasmid maintenance or selection, antibiotic concentration used was 10 µg/mL (Gm or Tet). The bacterial strains and plasmids used in this study are listed in Tables S1 to S3. Detailed descriptions of the strain and plasmid construction procedures can be found in the Supplementary Material.

### *P. aeruginosa* viability assay

For viability assays, overnight cell cultures were normalized to an OD_600_ of 1 and subjected to serial 10-fold dilutions with LB. Five microliters of each dilution was then spotted onto the indicated agar, and plates were incubated at 30°C for 24 h prior to imaging.

### *P. aeruginosa* electroporation

*P. aeruginosa* strains were made competent using previously described methods (53). Briefly, 4 mL of overnight cultures grown at 37°C were centrifuged and washed twice with 1-mL 300-mM sucrose. Cell pellets were resuspended in 500 µL of 300-mM sucrose, and 100 µL was used for electroporation. One microliter of replicative plasmid was used for the electroporation, using the following settings: 25 mF, 200 O, 2.5 kV. LB medium (1 mL) was added, and the cells were incubated with shaking (200 rpm) for 1 h at 37°C. Cells were then plated on the appropriate selective medium.

### Screen for mutants that induce *ampC* expression

The screening procedure was described previously (42). Briefly, *P. aeruginosa* strain CF263 [PAO1 (P*_ampC_::lacZ*)] was transposon-mutagenized by mating with the *E. coli* donor SM10(λpir) harboring a mariner transposon delivery vector pIT2 (54). The transposon confers Tet resistance. Mating mixtures were plated on LB agar supplemented with Tet (50 µg/mL) to select for transposon mutants and nalidixic acid (25 µg/mL) to select against the *E. coli* donor. The resulting collection of colonies was resuspended in LB broth and stored at −80°C. Dilutions of the library were plated on LB containing X-gal (50 µg/mL) to identify mutants with a constitutively active P*_ampC_::lacZ* reporter.

### Mapping of transposon insertion sites

Transposon insertions were mapped using arbitrarily primed PCR (54). Transposon-chromosomal junctions were amplified from mutant chromosomal DNA using the primers Rnd1-PA (5′-GGCCACGCGTCGACTAGTACNNNNNNNNNNGATAT-3′) and LacZ211 (5′-TGC GGG CCT CTT CGC TAT TA-3′). The resulting PCR reaction was used for a second PCR with primers Rnd2-PA (5′-GGCCACGCGTCGACTAGTAC-3′) and LacZ148 (5′-GGG TAA CGC CAG GGT TTT CC-3′). The final PCR product was sequenced using the transposon-specific primer LacZ-124L (5′-CAG TCA CGA CGT TGT AAA ACG ACC). The transposon-chromosomal DNA junction was identified in the sequencing reads using a nucleotide BLAST search (55) against the PAO1 genome (56).

### β-galactosidase assay

Beta-galactosidase assays were performed at room temperature. Cells from 100 µL of culture at OD_600_ = 0.1–0.6 were lysed with 30 µL of chloroform and mixed with 700 µL of Z buffer (60-mM Na_2_HPO_4_, 40-mM NaH_2_PO_4_, 10-mM KCl, and 1-mM MgSO_4_ heptahydrate). Each reaction then received 200 µL of o-nitrophenyl-β-D-galactopyranoside (ONPG, 4 mg/mL in 0.1-M KPO_4_ pH7.0), and the reaction was timed. After a medium-yellow color developed, the reaction was stopped with 400 µL of 1-M Na_2_CO_3_. The OD_420_ of the supernatant was determined, and the units of activity (Miller units) were calculated using the equation: U= (OD_420_ * 1,000) / [OD_660_ * time (in min) * volume of culture (in mL)].

### AmpC beta-lactamase activity assay

AmpC activity was assessed using nitrocefin hydrolysis. Overnight bacterial cultures were subcultured 1:20 in 3-mL LB or 6-mL LB supplemented with 1-mM IPTG and grown for 2 h at 30°C and 200 rpm. The cultures were split 1:1 in 2-mL LB with or without 50-µg/mL cefoxitin (final concentration), and all cultures were incubated for an additional 1.5 h at 30°C and 200 rpm. Following incubation, 1 mL of culture was pelleted at 2,300 × *g* for 5 min, washed once with 1 mL of 50-mM sodium phosphate buffer (pH 7.0), and resuspended in 1 mL of the same cold buffer. Samples were placed on ice and lysed at 4°C by sonication with a microprobe (Q800R2, QSonica, Newtown, Connecticut, USA). Sonicated samples were centrifuged at 12,000 × *g* for 5 min at 4°C, and supernatants were collected. The protein concentration was determined using a Bradford assay (57) with bovine serum albumin as the standard (G-Biosciences, Geno technology Inc., St. Louis, Missouri, USA). Nitrocefin hydrolysis assays were performed in 96-well plates. Each reaction had a final volume of 250 µL of 50-mM sodium phosphate buffer (pH 7.0) containing 10 µg of protein and 20 µg of nitrocefin (Thermo Fisher Scientific Oxoid, Waltham, Massachusetts, USA). Nitrocefin hydrolysis was monitored by measuring the absorbance at 486 nm every 5 min for 35 or 120 min at 30°C.

### Antibiotic sensitivity assays

Antibiotic sensitivity assays were performed using *E* test strips and broth microdilutions. For *E* test assays, 100 µL of bacterial cultures in exponential phase were evenly spread on LB agar plates and allowed to dry. *E* test strips were overlaid on the agar, and plates were incubated for 24 h at 37°C. The concentration at which the zone of inhibition intersected the *E* test strip was used to determine the minimum inhibitory concentrations (MICs). *E* test assays were performed three times independently, and the MIC values were listed in Table 1. For the antibiotic MIC assays, overnight cell cultures were normalized to OD_600_ of 0.0005 in LB and the indicated concentrations of A22, Carb, cefoxitin, mecillinam, or piperacillin and grown for 24 h at 30°C prior to taking optical density readings (Biotek Epoch 2, Agilent, Santa Clara, California, USA). Broth microdilution MIC assays were performed three or four times independently, each with two technical and two biological replicates.

### Intracellular soluble muropeptide analysis

To determine the presence and levels of intracellular soluble muropeptides, bacteria were grown until late exponential phase (roughly OD_600_ 0.7) in LB media before being cooled on ice for 10 min and normalized to the same OD_600_. Cells were then harvested by centrifugation at 10,000 × *g* for 10 min. The supernatant was discarded, and the cell pellet was washed three times in ice-cold 0.9% NaCl, resuspended in 0.9% NaCl so that the cells are 20 times concentrated and boiled for 10 min before centrifugation at maximum speed in a benchtop centrifuge for 10 min to remove the proteins and insoluble fraction. The supernatant was used for further analysis by liquid chromatography-mass spectrometry (LC-MS).

### PG isolation

Cells from 0.2-L cultures of overnight stationary phase were pelleted at 5,250 × *g* and resuspended in 5 mL of phosphate-buffered saline, added to an equal volume of 10% SDS in a boiling water bath and vigorously stirred for 3 h, and then stirred overnight at room temperature. The insoluble fraction (PG) was pelleted at 400,000 × *g*, 15 min, 30°C (TLA-100.3 rotor; Optima Max ultracentrifuge, Beckman) and resuspended in Milli-Q water. This step was repeated four to five times until the SDS was washed out. Next, PG was treated with Pronase E (0.1 mg/mL) at 60°C for 1 h and then boiled in 1% SDS for 2 h to stop the reaction. After SDS was removed as described previously, PG samples were resuspended in 200 µL of 50-mM sodium phosphate buffer pH 4.9 and digested overnight with 30-µg/mL muramidase (from *Streptomyces albus*) at 37°C. Muramidase digestion was stopped by heat inactivation (boiling for 5 min). Coagulated protein was removed by centrifugation (20,000 × *g*, 15 min). The supernatants (soluble muropeptides) were subjected to sample reduction. First, pH was adjusted to 8.5–9 by addition of borate buffer (0.5 M pH 9), and then muramic acid residues were reduced by sodium borohydride treatment (NaBH_4_ 10-mg/mL final concentration) during 30 min at room temperature. Finally, pH was adjusted to 2.0–4.0 with 25% orthophosphoric acid prior to analysis by LC.

### LC-MS analysis

Chromatographic analyses of muropeptides were performed by Ultra Performance Liquid Chromatography (UPLC) on an UPLC system (Waters) equipped with a trapping cartridge precolumn (SecurityGuard ULTRA Cartridge UHPLC C18 2.1 mm, Phenomenex) and an analytical column BEH C18 column (130 Å, 1.7 µm, 2.1 mm, Waters) maintained at 45°C. Muropeptides were detected by measuring the absorbance at 204 nm using an ACQUITY UPLC UV-visible detector. Muropeptides were separated using a linear gradient from Buffer A (water + 0.1% (vol/vol) formic acid) to Buffer B (acetonitrile 100% (vol/vol) + 0.1% (vol/vol) formic acid) over 15 min with a flow rate of 0.25 mL/min. The QTOF instrument was operated in positive ion mode, with data collection performed in untargeted MS^e^ mode. The parameters were set as follows: capillary voltage 3.0 kV, source temperature 120°C, desolvation temperature 350°C, sample cone voltage 40 V, cone gas flow 100 L h^−1^, and desolvation gas flow 500 L h^−1^. Mass spectra were acquired at a speed of 0.25 s/scan. The scan was in a range of 100–2,000 *m*/*z*. Data acquisition and processing were performed using MassLynx or UNIFI software package (Waters Corp.). The quantification of muropeptides was based on their relative abundances (relative area of the corresponding peak) and relative molar abundances. A table of all the identified muropeptides and the observed ions is provided (Table S4).

### Immunoblotting

Overnight bacterial cultures were subcultured 1:20 in 5-mL LB and grown for 4 h. Bacteria were collected by centrifugation, washed once with Tris–HCl buffer (pH = 8, 10 mM) and resuspended in 500 µL of the same cold buffer. The samples were then lysed at 4°C for 12 min at 60% amplitude with a pulse rate of 10 s ON/10 s OFF using a Qsonica sonicator. The samples were then centrifuged at 4°C at maximal speed to remove the cell debris. Supernatants were collected and a Bradford assay performed to measure the protein concentration. A total of 100-µg protein in a volume of 100 µL was mixed with 100 µL of 2× Laemmli buffer. Immunoblotting was performed by first separating 15 µL of each sample on 12% SDS-PAGE (polyacrylamide gel electrophoresis) gels at 90 V for 15 min and 120 V for an hour. Proteins were transferred at 90 V for an hour at 4°C to a 0.2-µm polyvinylidene difluoride membranes (Whatman) previously soaked in methanol and rinsed with transfer buffer. Membranes were blocked using 5% (wt/vol) skim milk in Tris-buffered saline (10-mM Tris–HCl pH 7.5, 150-mM NaCl) supplemented with 0.1% (vol/vol) Tween-20 (TBS-T) for 1 h. Membranes were incubated for 1 h with α-AmpC primary antibody (1:1,000 dilution in 5% skim milk in TBS-T, MyBioSource, MBS1493275, San Diego, USA) or α-FLAG primary antibody (1:1,000 dilution in 5% skim milk in TBS-T, F7425, Sigma-Aldrich) at 4°C. The membranes were washed four times in TBS-T for 5 min each before incubation for 1 h with secondary antibody (anti-rabbit IgG HRP, 1:5,000 dilution, Rockland 18–8816-33) in TBS-T with 5% (wt/vol) skim milk powder. The membranes were then washed four times with TBS-T for 5 min each before developing using SuperSignal West Pico PLUS Chemiluminescent Substrate (Thermo Fisher Scientific cat#34577) and imaged using the c600 Azure Biosystems platform.

## SUPPLEMENTAL MATERIAL

Supplemental material mbio.01419-24-s0001.docx Fig. S1 to S4, Tables S1 to S4, and additional experimental details.

ASM does not own the copyrights to Supplemental Material that may be linked to, or accessed through, an article. The authors have granted ASM a non-exclusive, world-wide license to publish the Supplemental Material files. Please contact the corresponding author directly for reuse.
