# Supplementary material for: A potential space-making role in cell wall biogenesis for SltB1and DacB revealed by a beta-lactamase induction phenotype in Pseudomonas aeruginosa
Source: mBio. 2024 Jun 26;15(7):e01419-24. doi: 10.1128/mbio.01419-24 (PMC11253642; doi:10.1128/mbio.01419-24)
Supplement: Supplemental material — Fig. S1 to S4, Tables S1 to S4, and additional experimental details. [file mbio.01419-24-s0001.docx]

**Supplemental Material for:**

**A potential space making role in cell wall biogenesis for SltB1and DacB revealed by a beta-lactamase induction phenotype in *Pseudomonas aeruginosa***

Joël Gyger^1^, Gabriel Torrens^2^, Felipe Cava^2^, Thomas G. Bernhardt^3,4*^ and Coralie Fumeaux^1,4*^

^1^ Institute of Microbiology, Lausanne University Hospital and University of Lausanne, 1011 Lausanne, Switzerland

^2^ Department of Molecular Biology and Laboratory for Molecular Infection Medicine Sweden, Umeå Centre for Microbial Research, SciLifeLab, Umeå University, Umeå, Sweden

^3^Howard Hughes Medical Institute, Chevy Chase, MD 20815, USA

^4^Department of Microbiology, Harvard Medical School, Boston, MA, USA

*To whom correspondence should be addressed

Thomas G. Bernhardt

Harvard Medical School

Department of Microbiology

Boston, Massachusetts 02115, USA

e-mail: thomas_bernhardt@hms.harvard.edu

Coralie Fumeaux

Institute of Microbiology

Lausanne University Hospital

University of Lausanne

Lausanne, Switzerland 1011

e-mail: coralie.fumeaux@unil.ch

**Running head:** SltB1 and *ampC* induction in *Pseudomonas aeruginosa*

**Figure S1.** (**A**) and (**B**) Cultures of the strains PAO1 [WT] and CF1105 [∆*sltB1*], harboring an empty vector (pKHT103) or plasmids (pCF533 or pCF1193) encoding *sltB1* or the catalytically inactive *sltB1(E135A)* under control of the lac promoter (P*_lac_*) were diluted and 5 µl of each dilution was spotted onto LB agar with or without IPTG inducer and/or ceftazidime (10 µg/ml)(**A**) or piperacillin (10 µg/ml)(**B**) as indicated.

**Figure S2.** Assays of nitrocefin hydrolysis by cell lysates of the strains PAO1 [WT], CF155 [∆*dacB*], CF256 [∆*dacB*∆*mltG*] and CF1446 [∆*dacB*∆*slt*]. Data are the mean of three independent assays each for two or three biological replicates with the error bars indicating the standard error. A one-way ANOVA revealed that there was a statistically significant difference in nitrocefin hydrolysis level between at least two strains (F(3, 23) = 5166.74, *p* <0.00001). Tukey’s HSD Test for multiple comparisons found that the mean value of nitrocefin hydrolysis level was significantly different between WT and ∆*dacB*, WT and ∆*dacB* ∆*slt*, ∆*dacB* and ∆*dacB* ∆*mltG* and finally ∆*dacB* ∆*mltG* and ∆*dacB* ∆*slt*, as indicated by asterisks (*p*=0.01).

**Figure S3.** (**A**) Cultures of strains PAO1 [WT], CF1105 [∆*sltB1*], and CF1416 [∆*sltB1* ∆*mltG*] containing plasmids pKHT103 [vector control], pCF658 [P*_lac_*::*mltG*], or pCF1328 [P*_lac_*::*mltG(E217Q)*] were serially diluted and 5 µl of each dilution was spotted onto LB agar with or without ceftazidime (10 µg/ml) and/or IPTG (1 mM) as indicated. (**B**) Cultures of strains PAO1 [WT], CF155 [∆*dacB*], and CF256 [∆*dacB* ∆*mltG*] containing plasmids pKHT103 [vector control], pCF658 [P*_lac_*::*mltG*], or pCF1328 [P*_lac_*::*mltG(E217Q)*] were serially diluted and 5 µl of each dilution was spotted onto LB agar with or without ceftazidime (10 µg/ml) and/or IPTG (1 mM) as indicated. (**C**) and (**D**) Derivatives of the strains in (**A**) or (**B**) with the P*_ampC_*::*lacZ* reporter were assayed for β-galactosidase activity as in **Fig. 2C**. β-galactosidase activity in Miller Units was measured in liquid cultures of the indicated strains. Results shown are the average of 4 assays with 2 biological replicates per strain and the error bars represent the standard deviation. Paired sample t-tests were performed to compare levels of *ampC* expression between strains and for each strain grown in LB or LB supplemented with IPTG [1mM]. The pairs presenting a significant difference with a *p* < 0.01 are highlighted with asterisks.

**Figure S4.** Cultures of strains CF 612 [∆*ampC*], CF550 [∆*ampR*] and CF1410 [∆*mltG*] with plasmids pJN105 [vector control], pCF1009 [P*_ara_*::*sltB1-FLAG*], or pCF1010 [P*_ara_*::*sltB1(E135A)-FLAG*] were serially diluted and 5 µl of each dilution was spotted onto LB agar supplemented with arabinose (0.01%) and ceftazidime (7.5 µg/ml) or piperacillin (7.5 µg/ml) as indicated.

**Table S1. *Pseudomonas aeruginosa* strains used in this study.**

| **Strain** | **Genotype** | **Source/Reference** |
| --- | --- | --- |
| PAO1 | *Wild-type* | (1) |
| CF155 | PAO1 ∆*dacB (PA3047)* | (2) |
| CF406 | PAO1 *sltB1*::Tn | This study |
| CF612 | PAO1 ∆*ampC (PA4110)* | (2) |
| CF550 | PAO1 ∆*ampR (PA4109)* | (2) |
| CF1105 | PAO1 ∆*sltB1 (PA4001)* | This study |
| CF1410 | PAO1 ∆*mltG (PA2963)* | This study |
| CF918 | PAO1 ∆*slt (PA3020)* | This study |
| CF186 | PAO1 ∆*ampD (PA4522)* | This study |
| CF370 | PAO1 ∆*sltB1*∆*ampR* | This study |
| CF368 | PAO1 ∆*sltB1*∆*ampC* | This study |
| CF372 | PAO1 ∆*sltB1*∆*ampG* | This study |
| CF1416 | PAO1 ∆*sltB1*∆*mltG* | This study |
| CF378 | PAO1 ∆*sltB1*∆*slt* | This study |
| CF256 | PAO1 ∆*dacB*∆*mltG* | This study |
| CF1446 | PAO1 ∆*dacB*∆*slt* | This study |
| CF1585 | PAO1 ∆*sltB1*∆*ampD* | This study |
| CF1591 | PAO1 ∆*sltB1*∆*mltG*∆*ampD* | This study |
| CF189 | PAO1 ∆*dacB*∆*ampD* | This study |
| CF1588 | PAO1 ∆*dacB*∆*mltG*∆*ampD* | This study |
| CF732 | PAO1 *att*Tn7::*P_lac_::empty* | (2) |
| CF666 | PAO1 *att*Tn7::*P_lac_::sltB1* | This study |
| CF1122 | PAO1 ∆*sltB1 att*Tn7::*P_lac_::empty* | This study |
| CF1124 | PAO1 ∆*sltB1 att*Tn7::*P_lac_::sltB1* | This study |
| CF1212 | PAO1 ∆*sltB1 att*Tn7::*P_lac_::sltB1 E135A* | This study |
| CF1447 | PAO1 ∆*sltB1*∆*mltG att*Tn7::*P_lac_::empty* | This study |
| CF1449 | PAO1 ∆*sltB1*∆*mltG att*Tn7::*P_lac_::mltG* | This study |
| CF1451 | PAO1 ∆*sltB1*∆*mltG att*Tn7::*P_lac_::mltG E217Q* | This study |
| CF311 | PAO1 ∆*dacB att*Tn7::*P_lac_::empty* | This study |
| CF1437 | PAO1 ∆*dacB*∆*mltG att*Tn7::*P_lac_: :empty* | This study |
| CF1439 | PAO1 ∆*dacB*∆*mltG att*Tn7::*P_lac_::mltG* | This study |
| CF1441 | PAO1 ∆*dacB*∆*mltG att*Tn7::*P_lac_::mltG E217Q* | This study |
| CF262 | PAO1 *attB*::*P_ampC_ (352 bp)-lacZ* | (2) |
| CF268 | PAO1 ∆*dacB attB*::*P_ampC_ (352 bp)-lacZ* | (2) |
| CF604 | PAO1 ∆*ampR attB*::*P_ampC_ (352 bp)-lacZ* | (2) |
| CF1143 | PAO1 ∆*sltB1* *attB*::*P_ampC_ (352 bp)-lacZ* | This study |
| CF358 | PAO1 ∆*sltB1* ∆*ampR attB*::*P_ampC_ (352 bp)-lacZ* | This study |
| CF1422 | PAO1 ∆*sltB1* ∆*mltG attB*::*P_ampC_ (352 bp)-lacZ* | This study |
| CF263 | PAO1 ∆*dacB* ∆*mltG attB*::*P_ampC_ (352 bp)-lacZ* | This study |
| CF1304 | PAO1 ∆*sltB1* ∆*mltG attB*::*P_ampC_ (352 bp)-lacZ att*Tn7::*P_lac_::empty* | This study |
| CF1306 | PAO1 ∆*sltB1*∆*mltG attB*::*P_ampC_ (352 bp)-lacZ*  *att*Tn7::*P_lac_::mltG* | This study |
| CF1308 | PAO1 ∆*sltB1*∆*mltG attB*::*P_ampC_ (352 bp)-lacZ*  *att*Tn7::*P_lac_::mltG E217Q* | This study |
| CF1298 | PAO1 ∆*dacB*∆*mltG attB*::*P_ampC_ (352 bp)-lacZ att*Tn7::*P_lac_::empty* | This study |
| CF1300 | PAO1 ∆*dacB*∆*mltG attB*::*P_ampC_ (352 bp)-lacZ*  *att*Tn7::*P_lac_::mltG* | This study |
| CF1302 | PAO1 ∆*dacB*∆*mltG attB*::*P_ampC_ (352 bp)-lacZ*  *att*Tn7::*P_lac_::mltG E217Q* | This study |
| CF1631 | PAO1 (P_ara_-empty) | This study |
| CF1632 | PAO1 (P_ara_-*sltB1*-FLAG) | This study |
| CF1634 | PAO1 (P_ara_-*sltB1 E135A*-FLAG) | This study |
| CF1726 | PAO1 ∆*ampC* (P_ara_-empty) | This study |
| CF1727 | PAO1 ∆*ampC* (P_ara_-*sltB1*-FLAG) | This study |
| CF1728 | PAO1 ∆*ampC* (P_ara_-*sltB1 E135A*-FLAG) | This study |
| CF1731 | PAO1 ∆*ampR* (P_ara_-empty) | This study |
| CF1732 | PAO1 ∆*ampR* (P_ara_-*sltB1*-FLAG) | This study |
| CF1733 | PAO1 ∆*ampR* (P_ara_-*sltB1 E135A*-FLAG) | This study |
| CF1738 | PAO1 ∆*mltG* (P_ara_-empty) | This study |
| CF1739 | PAO1 ∆*mltG* (P_ara_-*sltB1*-FLAG) | This study |
| CF1740 | PAO1 ∆*mltG* (P_ara_-*sltB1 E135A*-FLAG) | This study |
| CF1638 | PAO1 ∆*sltB1* (P_ara_-empty) | This study |
| CF1639 | PAO1 ∆*sltB1* (P_ara_-*sltB1*-FLAG) | This study |
| CF1641 | PAO1 ∆*sltB1* (P_ara_-*sltB1 E135A*-FLAG) | This study |

**Table S2. *Escherichia coli* strains used in this study.**

| **Strain** | **Genotype^a^** | **Source/Reference^b^** |
| --- | --- | --- |
| DH5α | *F– hsdR17 deoR recA1 endA1 phoA supE44 thi-1 gyrA96 relA1 Δ(lacZYA-argF)U169* ϕ*80dlacZΔM15* | Gibco BRL |
| Sm10(λpir) | *Kan^R^ thi-1 thr leu tonA lacY supE recA::RP4-2-Tc::Mu attλ::pir* | (3) |

**Table S3.** Plasmids used in this study.

| ***Plasmid*** | ***Genotype*** | ***ori*** | ***Source/Reference*** |
| --- | --- | --- | --- |
| pEXG2 | *aacC1 sacB oriT [vector for allelic exchange in P. aeruginosa]* | pBR/colE1 | (4) |
| pFLP2 | *bla sacB flp cI oriT [plasmid for Flp recombinase expression]* | pRO1600 | (5) GenBank no. AF048702 |
| pKHT103 | *aacC1 bla* Tn7 *lacI^q^ Plac [vector for insertion of Plac-regulated sequences into the att*Tn7 *attachment site of P. aeruginosa]* | pUC18 | Dove lab |
| pTNS2 | *bla oriR6K tnsABCD [plasmid for* Tn7 *transposase expression]* | R6K | (6) |
| pJN105 | *aacC1 araC* P_ara_ *[replicating arabinose-inducible expression vector for P. aeruginosa]* | pBBR1 | (7) |
| pOK12 | *Kan^R^ lacZ* | p15A | (8) |
| pCF533 | *aacC1 bla* Tn7 *lacI^q^ Plac::sltB1* | pUC18 | This study |
| pCF1193 | *aacC1 bla* Tn7 *lacI^q^ Plac::sltB1 E135A* | pUC18 | This study |
| pCF658 | *aacC1 bla* Tn7 *lacI^q^ Plac::mltG* | pUC18 | This study |
| pCF1328 | *aacC1 bla* Tn7 *lacI^q^ Plac::mltG E217Q* | pUC18 | This study |
| pCF579 | *aacC1 sacB oriT ‘PA4110-ampC∆ (3-289)’* | pBR/colE1 | (2) |
| pCF583 | *aacC1 sacB oriT ‘PA4109-ampR∆ (6-294)’* | pBR/colE1 | (2) |
| pCF198 | *aacC1 sacB oriT ‘PA3047-dacB∆ (1-476)’* | pBR/colE1 | (2) |
| pCF284 | *aacC1 sacB oriT ‘PA4393-ampG∆ (1-594)’* | pBR/colE1 | (2) |
| pCF1097 | *aacC1 sacB oriT ‘PA4001-sltB1∆ (193-338)’* | pBR/colE1 | This study |
| pCF1258 | *aacC1 sacB oriT ‘PA2963-mltG∆ (45-207)’* | pBR/colE1 | This study |
| pCF856 | *aacC1 sacB oriT ‘PA3020-slt∆ (3-635)’* | pBR/colE1 | This study |
| pCF696 | *aacC1 sacB oriT ‘PA4522-ampD∆ (2-187)’* | pBR/colE1 | This study |
| pOC1 | *P_ampC(352 bp)_* fused to the *lacZ* of mini-CTX-*lacZ* | pBR/colE1 (pMB1) | (9) |
| pCF1175 | *Kan^R^ lacZ -sltB1 (pOK12 derivative)* | p15A | This study |
| pCF1189 | *Kan^R^ lacZ -sltB1 E135A (pOK12 derivative)* | p15A | This study |
| pCF1009 | *aacC1 araC* P_ara_:: RBS_optimized_-*sltB1-FLAG* | pBBR1 | This study |
| pCF1010 | *aacC1 araC* P_ara_:: RBS_optimized_-*sltB1 E135A-FLAG* | pBBR1 | This study |

**Table S4.** Identified muropeptides.

| **Identity** | **Proposed Structure** | **Observed *m/z* value** | **Observations** |
| --- | --- | --- | --- |
| M3 | GlcNAc-MurNAc-L-Ala-D-Glu-m-DAP | 871.3784 | [M+H]+ |
| M3G | GlcNAc-MurNAc-L-Ala-D-Glu-m-DAP-Gly | 928.3992 | [M+H]+ |
| M4 | GlcNAc-MurNAc-L-Ala-D-Glu-m-DAP-D-Ala | 942.4156 | [M+H]+ |
| D33 | M3-M3 (DAP-m-DAP crosslink) | 862.3771 | [M+2H]2+ |
| D43G | M4-M3G (D-Ala-m-DAP crosslinks) | 926.4024 | [M+2H]2+ |
| D43 | M4-M3 (D-Ala-m-DAP crosslink) | 897.8920 | [M+2H]2+ |
| D43^NH2^ | M4-M3^NH2^ (D-Ala-m-DAP crosslink) | 897.3997 | [M+2H]2+ |
| D44 | M4-M4 (D-Ala-m-DAP crosslink) | 933.4114 | [M+2H]2+ |
| T444 | M4-M4-M4 (D-Ala-m-DAP crosslink) | 930.4082 | [M+3H]3+ |
| T444^Anh^ | M4-M4-M4^Anh^ (D-Ala-m-DAP crosslink) | 923.7331 | [M+3H]3+ |
| UDP-M5 | UDP-MurNAc-L-Ala-D-Glu-m-DAP-D-Ala-D-Ala | 1194.3492 | [M+H]+ |
| NAM^Anh^-P3 | Anhydro-MurNAc-L-Ala-D-Glu-m-DAP | 648.2728 | [M+H]+ |
| NAM^Anh^-P5 | Anhydro-MurNAc-L-Ala-D-Glu-m-DAP-D-Ala-D-Ala | 790.3470 | [M+H]+ |

*GlcNAc: N-acetylglucosamine; MurNAc: N-acetylmuramic acid (NAM); Anh: anhydro N-acetylmuramic acid; L/D-Ala: alanine; D-Glu: glutamic acid; m-DAP: meso-diaminopimelic acid. NH_2_: amidated L-center of m-DAP.

**Plasmid construction:**

For all plasmid constructions (**Table S3**), PCR was performed using Phusion or the Q5 polymerase (New England Biolabs) according to the manufacturer’s instructions. Plasmid DNA was purified using the Zippy miniprep kits (Zymo Research) while PCR fragments were purified using a Qiaquick PCR purification kit (Qiagen). Unless otherwise indicated, plasmids were constructed using the Gibson isothermal assembly method (10) and the reactions were incubated at 50°C for 30 minutes.

To construct **pCF696 [***aacC1 sacB oriT ‘PA4522-ampD∆ (2-187)’*], which is used for deletion of *ampD* (*PA4522*), the ~800bp region upstream of *ampD* was amplified from PAO1 genomic DNA (gDNA) using 5’- AAA TGT AAA GCA AGC TTC TGC AGG TCG ACT CT ATA CCA TGG ACG AGC TGA TCT GGA GCG TCG -3’ and 5’- AAG GGC CAG CAG CAA CAC CAG GAA CGT CA CAT ACG GGC TCC TGA ACG AGC CCC CAC TCT -3’ primers. The ~800bp region downstream of the gene was amplified with 5’- AGA GTG GGG GCT CGT TCA GGA GCC CGT ATG TGA CGT TCC TGG TGT TGC TGC TGG CCC TT -3’ and 5’- GAA TTC GAG CTC GAG CCC GGG GAT CCT CTA ACC AGC AAT TGC CAG AGC GCA TCC AGG CT -3’ primers. The two resulting fragments were then combined by isothermal assembly into pEXG2, which was previously digested with XbaI.

To construct **pCF856** [*aacC1 sacB oriT ‘PA3020-slt∆(3-635)’*], which is used for deletion of *slt* (*PA3020*), the ~800bp region upstream of *slt* was amplified from PAO1 genomic DNA (gDNA) using 5’-AAA TGT AAA GCA AGC TTC TGC AGG TCG ACT CTT TGG TCG GTT CGT CGA GCA GCA GCA GGT-3’ and 5’-TTG GGG GTC AGA AAT CGT CGA AGT AGC GTT CGC GCA TGA CTT ATC CGG GCA GGT GAT GT-3’ primers. The ~800bp region downstream of the gene was amplified with 5’-ACA TCA CCT GCC CGG ATA AGT CAT GCG CGA ACG CTA CTT CGA CGA TTT CTG ACC CCC AA-3’ and 5’-GAA TTC GAG CTC GAG CCC GGG GAT CCT CTA TCG AGT TCG TCG TGG CCA AGC CCT GTT-3’ primers. The two resulting fragments were then combined by isothermal assembly into pEXG2, which was previously digested with XbaI.

To construct **pCF1097** [*aacC1 sacB oriT ‘PA4001-sltB1∆(193-338)’*], which is used for deletion of *sltB1* (*PA4001*), the ~800bp region upstream of *sltB1* was amplified from PAO1 genomic DNA (gDNA) using 5’-AAA TGT AAA GCA AGC TTC TGC AGG TCG ACT CTA GTT CAT GAA GCT GCT GAT GCC GAT GA-3’ and 5’-ATC AGG GAG GAG CGG ACA CGC TTG CTC AAT GGG CCG CCG GCG TAG GAG CCG GTC AGG CTG A-3’ primers. The ~800bp region downstream of the gene was amplified with 5’-TCA GCC TGA CCG GCT CCT ACG CCG GCG GCC CAT TGA GCA AGC GTG TCC GCT CCT CCC TGA T-3’ and 5’-GAA TTC GAG CTC GAG CCC GGG GAT CCT TTT TTT TTT GAG TCG ATC TGC ACC GGC AGC-3’ primers. The two resulting fragments were then combined by isothermal assembly into pEXG2, which was previously digested with XbaI.

To construct **pCF1258** [*aacC1 sacB oriT ‘PA2963-mltG∆(45-207)’*], which is used for deletion of *mltG* (*PA2963*), the ~800bp region upstream of *mltG* was amplified from PAO1 genomic DNA (gDNA) using 5’-AAA TGT AAA GCA AGC TTC TGC AGG TCG ACT CTA CTG GCC TAT GGC GAC GGG CTG TTC GAG A-3’ and 5’-CTT TTC CAC CAG CGA GGC CAT GAT CAG AAC GTC CAG CAG GCG CTC CTC GGT CAA TTG-3’ primers. The ~800bp region downstream of the gene was amplified with 5’-CAA TTG ACC GAG GAG CGC CTG CTG GAC GTT CTG ATC ATG GCC TCG CTG GTG GAA AAG-3’ and 5’-GAA TTC GAG CTC GAG CCC GGG GAT CCT AAT GCT CGG CCA GGG CAC GCT TAC CGA T-3’ primers. The two resulting fragments were then combined by isothermal assembly into pEXG2, which was previously digested with XbaI.

**pCF533** [*aacC1 bla Tn7* *lacI^q^* P*_lac_*::*sltB1*]: For the construction of pCF533 for integration of an IPTG-inducible version of *sltB1* at the Tn7 attachment (*att*Tn7) site, *sltB1* was amplified from PAO1 genomic DNA using 5’-AGC TTA GTC GAC AGC TAG CCG GAT CCC CGG GAG GAG GAT ACA TGT GAA GAA CGC AAT GCA AGT ACT GCG TAC-3’ and 5’-AAG GGG TTA TGC TAA AGC TTG CAT GCG GTA CTC AAT GGG CAC CTC GCG CGC GGG CAA TCT-3’ primers. The optimized RBS is underlined. The resulting fragment was then inserted by Gibson isothermal assembly into a KpnI-digested expression vector pKHT103 to generate pCF533.

**pCF658** [*aacC1 bla Tn7* *lacI^q^* P*_lac_*::*mltG*]: For the construction of pCF658 for integration of an IPTG-inducible version of *mltG* at the Tn7 attachment (*att*Tn7) site, *mltG* was amplified from PAO1 genomic DNA using 5’-AGC TTA GTC GAC AGC TAG CCG GAT CCC CGG GAG GAG GAT ACA TAT GCG CAA ACT GCT GGT GCT GCT GGA GAG-3’ and 5’-AAG GGG TTA TGC TAA AGC TTG CAT GCG GTA CTC ATT GTG GCG GCG GGG TGA TGG GC-3’ primers. The optimized RBS is underlined. The resulting fragment was then inserted by Gibson isothermal assembly into a KpnI-digested expression vector pKHT103 to generate pCF658.

Site-directed mutagenesis was performed using the QuikChange method (Stratagene) or PCR site directed mutagenesis.

For **pCF1175** [*Kan^R^ lacZ -sltB1* (pOK12 derivative)], the *sltB1* gene was amplified from wild-type genomic DNA using the primers *sltB1* HindIII 5’ optimized RBS (5’- AAA AAG CTT **GAG GAG GAT ACA T**GT GAA GAA CGC AAT GCA AGT ACT GCG TAC -3’) and *sltB1* XbaI 3’ (5’- AAA TCT AGA TCA ATG GGC ACC TCG CGC GCG GGC AAT CT -3’). The restriction sites are underlined and the optimized RBS is bolded. The PCR product was then digested with XbaI and HindIII and ligated into XbaI/HindIII digested pOK12. The primer sequences used for the mutagenesis are *sltB1 E135A* #1 (5’- ATC ATC GGC GTG GCA ACC TTC TTC GGC -3’) and *sltB1 E135A* #2 (5’- GCC GAA GAA GGT TGC CAC GCC GAT GAT -3’). The PCR was performed using KOD polymerase (Novagen) according to the manufacturer’s instructions (65˚C annealing temperature and 2.5 minutes of extension for 20 cycles). The PCR product was directly treated for 5 hours at 37˚C with 1 µl of DpnI restriction enzyme to digest the parental double-stranded DNA. A portion of the reaction (5 µl) was used to transform chemo-competent DH5α and transformants were selected on LB plates containing 25 µg/ml kanamycin. The plasmid with the correct mutation was identified by sequencing and was designated **pCF1189** [*Kan^R^ lacZ-sltB1 E135A* (pOK12 derivative)].

**pCF1193** [*aacC1 bla Tn7* *lacI^q^ P_lac_::sltB1 E135A*]. For the construction of pCF1193 for integration of an IPTG-inducible version of *sltB1(E135A)* at the Tn7 attachment (*att*Tn7) site, *sltB1(E135A)* was amplified from pCF1189 [*Kan^R^ lacZ-sltB1 E135A* (pOK12 derivative)] plasmid DNA using 5’-AGC TTA GTC GAC AGC TAG CCG GAT CCC CGG GAG GAG GAT ACA TGT GAA GAA CGC AAT GCA AGT ACT GCG TAC-3’ and 5’-AAG GGG TTA TGC TAA AGC TTG CAT GCG GTA CTC AAT GGG CAC CTC GCG CGC GGG CAA TCT-3’ primers. The optimized RBS is underlined. The resulting fragment was then inserted by Gibson isothermal assembly into a KpnI-digested expression vector pKHT103 to generate pCF1193.

**pCF1328** [*aacC1 bla Tn7* *lacI^q^ P_lac_::mltG E217Q*]. The plasmid pCF1328 for integration of an IPTG-inducible version of *mltG(E217Q)* catalytic mutant at the Tn7 attachment (*att*Tn7) site was created in several steps. First, the 5’ end of *mltG* gene was PCR-amplified from PAO1 gDNA with 5’-AGC TTA GTC GAC AGC TAG CCG GAT CCC CGG GAG GAG GAT ACA TAT GCG CAA ACT GCT GGT GCT GCT GGA GAG-3’ and *mltG E217Q* #2 (5’-TTC CGG CAC GCC GGT TTG CTT TTC CAC CAG CG-3’) primers. Concurrently, the 3’ end of *mltG* gene was PCR-amplified from PAO1 gDNA with *mltG E217Q* #1 (5’-CGC TGG TGG AAA AGC AAA CCG GCG TGC CGG AA-3’) and 5’-AAG GGG TTA TGC TAA AGC TTG CAT GCG GTA CTC ATT GTG GCG GCG GGG TGA TGG GC-3’ primers. The optimized RBS and the mutated site are underlined. These two PCR products were then combined by sewing PCR with 5’-AGC TTA GTC GAC AGC TAG CCG GAT CCC CGG GAG GAG GAT ACA TAT GCG CAA ACT GCT GGT GCT GCT GGA GAG-3’ and 5’-AAG GGG TTA TGC TAA AGC TTG CAT GCG GTA CTC ATT GTG GCG GCG GGG TGA TGG GC-3’ primers. This final PCR product was then inserted by Gibson isothermal assembly into a KpnI-digested expression vector pKHT103 to generate pCF1328.

For **pCF1009** [*aacC1 araC* P_ara_:: RBS_optimized_-*sltB1-FLAG*] and **pCF1010** [P_ara_:: RBS_optimized_-*sltB1 E135A-FLAG*], the *sltB1* genes were amplified from PAO1 gDNA or the appropriate mutated versions (pCF1193) using 5’-AAT TCC TGC AGC CCG GGG GAT CCA CTA GTT GAG GAG GAT ACA TGT GAA GAA CGC AAT GCA AGT ACT GCG TAC-3’ and 5’-TTG GAG CTC CAC CGC GGT GGC GGC CGC TCT AG**T CAC TTA TCA TCA TCA TCC TTA TAG TC**A TGG GCA CCT CGC GCG CGG GCA ATC T-3’ primers. The optimized RBS is underlined and the FLAG tag sequence is bolded. The resulting fragments were then inserted by Gibson isothermal assembly into a XbaI-digested expression vector pJN105 (7).

***P. aeruginosa* strain construction.**

Briefly, during *P. aeruginosa* strain construction, plasmids were transferred into *P. aeruginosa* by conjugation from an *E. coli* donor [SM10(λpir)] on LB plates. Counter-selection against *E. coli* was accomplished on Vogel-Bonner minimal medium (VBMM)(11) supplemented with 30 μg/ml gentamicin.

To create the Δ*sltB1* strains CF1105 [PAO1∆*sltB1*] and CF1143 [PAO1∆*sltB1 attB::*P*_ampC_-lacZ*], pCF1097 [*aacC1 sacB oriT* *‘PA4001-sltB1*∆*(193-338)’*] was conjugated into PAO1 [WT] and CF263 [PAO1 *attB::*P*_ampC_-lacZ*] recipient from SM10(λpir) donor. For this purpose, PAO1 and CF263 were patched on an LB plate and grown overnight at 42°C while SM10(λpir) carrying pCF1097 was similarly grown at 37°C. Both the donor and the recipients were scraped, patched together onto an LB plate, and incubated at 37°C for ~5h. The cells were scraped, resuspended in 500 μL of VBMM, diluted 1:10, and 100 μL of the resulting suspension was plated on VBMM supplemented with 30 μg/mL gentamicin. Plates were incubated at 37°C overnight. The exconjugants were purified on LB supplemented with 30 μg/mL gentamicin. A few single colonies were allowed to grow for ~6h in plain LB broth to allow for the second plasmid recombination event, and 100 μL of the resulting culture was plated on LB supplemented with 5% (w/v) sucrose to select for the loss of the plasmid-encoded *sacB* gene. Sucrose-resistant colonies were then patched onto LB plates either containing or lacking 30 μg/mL gentamicin. Gentamicin-sensitive colonies were further tested by PCR with *sltB1*-flanking primers 5’-AAA TGT AAA GCA AGC TTC TGC AGG TCG ACT CTA GTT CAT GAA GCT GCT GAT GCC GAT GA-3’ and 5’-GAA TTC GAG CTC GAG CCC GGG GAT CCT TTT TTT TTT GAG TCG ATC TGC ACC GGC AGC-3’ to confirm gene deletion. The deletion retains the first one hundred ninety-two and last two codons of the *sltB1* reading frame. This truncation is similar to the one described in the previous study by Cavallari *et al*., which carries a SCAR mutation at nucleotide 577 of *sltB1* (12).

To create the Δ*slt* strains CF918 [PAO1∆*slt*], CF378 [PAO1∆*sltB1*∆*slt*] and CF1446 [PAO1∆*dacB*∆*slt*], *slt* was deleted from PAO1 [WT], CF1105 [PAO1∆*sltB1*] and CF155 [PAO1∆*dacB*] by integration and re-circularization of pCF856 [*aacC1 sacB oriT* *‘PA3020-slt*∆*(3-635)’*] as described above. Sucrose-resistant, gentamicin-sensitive colonies were screened by PCR with *slt*-flanking primers 5’-AAC AGG CTG GAC TTG CCG GTA CCG TT-3’ and 5’-TTT TCT GGC CGT CGT TCA GGT GCT-3’. The deletion retains the first two and last eight codons of the *slt* reading frame.

To create the Δ*mltG* strains CF1410 [PAO1∆*mltG*], CF1416 [PAO1∆*sltB1*∆*mltG*], CF1422 [PAO1∆*sltB1*∆*mltG* attB*::*P*_ampC_-lacZ*], CF256 [PAO1∆*dacB*∆*mltG*] and CF263 [PAO1∆*dacB*∆*mltG* attB*::*P*_ampC_-lacZ*], *mltG* was deleted from PAO1 [WT], CF1105 [PAO1∆*sltB1*], CF1143 [PAO1∆*sltB1 attB::*P*_ampC_-lacZ*], CF155 [PAO1∆*dacB*] and CF268 [PAO1∆*dacB attB::*P*_ampC_-lacZ*] by integration and re-circularization of pCF1258 plasmid [*aacC1 sacB oriT* ‘*PA2963*-*mltG*∆*(45-207)’*] as described above. The sucrose-resistant, gentamicin-sensitive colonies were screened by PCR with *mltG*-flanking primers 5’-AAA TGT AAA GCA AGC TTC TGC AGG TCG ACT CTA CTG GCC TAT GGC GAC GGG CTG TTC GAG A-3’ and 5’-GAA TTC GAG CTC GAG CCC GGG GAT CCT AAT GCT CGG CCA GGG CAC GCT TAC CGA T-3’. The deletion retains the first fourty-four and last one hundred fourty-three codons of the *mltG* reading frame.

To create the Δ*ampR* strains CF370 [PAO1∆*sltB1*∆*ampR*] and CF358 [PAO1∆*sltB1*∆*ampR attB::*P*_ampC_-lacZ*], *ampR* was deleted from CF1105 [PAO1∆*sltB1*] and CF1143 [PAO1∆*sltB1 attB::*P*_ampC_-lacZ*] by integration and re-circularization of pCF583 plasmid [*aacC1 sacB oriT* ‘*PA4109 ampR*∆*(6-294)’*] as described above (2). Sucrose-resistant, gentamicin-sensitive colonies were screened by PCR with *ampR*-flanking primers 5’- AAC ACT TGC TGC TCC ATG AGC CGT TCG AA -3’ and 5’- AAG GTA TTC TTC TCG GCC CGC TCG AAG GT -3’. The deletion retains the first five and the last three codons of the *ampR* reading frame.

To create the Δ*ampG* strain CF372 [PAO1∆*sltB1*∆*ampG*], *ampG* was deleted from CF1105 [PAO1∆*sltB1*] by integration and re-circularization of pCF284 plasmid [*aacC1 sacB oriT ‘PA4393-ampG*∆*(1-594)’*] as described above (2). Sucrose-resistant, gentamicin-sensitive colonies were screened by PCR with *ampG*-flanking primers 5’- TAG AGC GGT TAG AGT GCG CGT TA-3’ and 5’- GTG CGA TCC ACG AAA AAG GC-3’. The deletion does not retain any *ampG* sequence.

To create the Δ*ampC* strain CF368 [PAO1∆*sltB1*∆*ampC*], *ampC* was deleted from CF1105 [PAO1∆*sltB1*] by integration and re-circularization of pCF579 plasmid [*aacC1 sacB oriT ‘PA4110-ampC*∆*(3-289)’*] as described above (2). Sucrose-resistant, gentamicin-sensitive colonies were screened by PCR with *ampC*-flanking primers 5’- ATG TCG ACG CGG TTG TTG TGG GTG GAC A -3’ and 5’- ATG GAA ATC CTC GCC GGC ATC CGC CTC -3’. The deletion retains the first three and the last nine codons of *ampC*.

To create the Δ*ampD* strains CF186 [PAO1∆*ampD*], CF1585 [PAO1∆*sltB1*∆*ampD*], CF1591 [PAO1∆*sltB1*∆*mltG*∆*ampD*], CF189 [PAO1∆*dacB*∆*ampD*] and CF1589 [PAO1∆*dacB*∆*mltG*∆*ampD*], *ampD* was deleted from PAO1 [WT], CF1105 [PAO1∆*sltB1*], CF1416 [PAO1∆*sltB1*∆*mltG*], CF155 [PAO1∆*dacB*] and CF256 [PAO1∆*dacB*∆*mltG*] by integration and re-circularization of pCF696 [*aacC1 sacB oriT ‘PA4522-ampD∆ (2-187)’*] as described above. Sucrose-resistant, gentamicin-sensitive colonies were screened by PCR with *ampD*-flanking primers 5’- AAG GTC CTG GAA AAG ACC CGC ATG G -3’ and 5’- AAC GAC TGC AGC AAT GTC AGC AAC AGG -3’. The deletion retains the first two and the last codon of the *ampD* reading frame.

Construction of strains CF666 [PAO1 *att*Tn7::*P_lac_::sltB1*] and CF1124 [PAO1 ∆*sltB1 att*Tn7::*P_lac_::sltB1*] with a P_lac_-regulated copy of *sltB1* (P*_lac_*::*sltB1*) integrated at Tn7 locus was based on a previously described protocol (11). In brief, plasmid pCF533, which encodes a P*_lac_*-regulated copy of *sltB1* flanked by Tn7 transposon inverted repeats, and plasmid pTNS2, which encodes Tn7 transposase, were co-electroporated into PAO1 [WT] and CF1105 [PAO1∆*sltB1*]. Transformants were selected on LB plates supplemented with 30 μg/mL gentamicin. The integration of the transposon at the Tn7 attachment locus was confirmed by diagnostic PCR with PTn7R and PglmS-down primers (11). The gentamicin resistance cassette was then removed by Flp-mediated excision. Plasmid pFLP2 was electroporated and transformants were selected for growth on LB medium supplemented with 200 μg/mL carbenicillin, as described previously (11). Carbenicillin-resistant transformants were patched onto plain LB agar or LB supplemented with gentamicin to confirm the loss of the gentamicin resistance cassette. Gentamicin sensitive clones were grown overnight in liquid LB medium lacking antibiotics and purified on LB agar supplemented with 5% (w/v) sucrose to select for the loss of the pFLP2 plasmid, which encodes the *sacB* gene. Isolated colonies were patched onto LB supplemented with carbenicillin or no antibiotic to confirm the loss of the pFLP2 plasmid. The construction of strain CF1122 [PAO1 ∆*sltB1 att*Tn7::*P_lac_::empty*] and CF1212 [PAO1 ∆*sltB1 att*Tn7::*P_lac_::sltB1 E135A*], with P*_lac_*::empty and P*_lac_*::*sltB1* integrated at the Tn7 locus were performed as above in strain CF1105 [PAO1∆*sltB1*], but using plasmids pKHT103 and pCF1193, respectively.

Construction of strains CF1447 [PAO1 ∆*sltB1*∆*mltG att*Tn7::*P_lac_::empty*], CF1449 [PAO1 ∆*sltB1*∆*mltG att*Tn7::*P_lac_::mltG*] and CF1451 [PAO1 ∆*sltB1*∆*mltG att*Tn7::*P_lac_::mltG E217Q*] with P*_lac_*::empty, P*_lac_*::*mltG* and P*_lac_*::*mltG E217Q* integrated at the Tn7 locus were performed as above in strain CF1416 [PAO1∆*sltB1*∆mltG] using plasmids pKHT103, pCF658 and pCF1328, respectively.

Construction of strains CF1437 [PAO1 ∆*dacB*∆*mltG att*Tn7::*P_lac_::empty*], CF1439 [PAO1 ∆*dacB*∆*mltG att*Tn7::*P_lac_::mltG*] and CF1441 [PAO1 ∆*dacB*∆*mltG att*Tn7::*P_lac_::mltG E217Q*] with P*_lac_*::empty, P*_lac_*::*mltG* and P*_lac_*::*mltG E217Q* integrated at the Tn7 locus were performed as above in strain CF1412 [PAO1∆*dacB*∆mltG] using plasmids pKHT103, pCF658 and pCF1328, respectively.

Construction of strains CF1304 [PAO1 ∆*sltB1*∆*mltG attB::*P*_ampC_-lacZ att*Tn7::*P_lac_::empty*], CF1306 [PAO1 ∆*sltB1*∆*mltG attB::*P*_ampC_-lacZ att*Tn7::*P_lac_::mltG*] and CF1308 [PAO1 ∆*sltB1*∆*mltG attB::*P*_ampC_-lacZ att*Tn7::*P_lac_::mltG E217Q*] with P*_lac_*::empty, P*_lac_*::*mltG* and P*_lac_*::*mltG E217Q* integrated at the Tn7 locus were performed as above in strain CF1422 [PAO1∆*sltB1*∆*mltG* *attB::*P*_ampC_-lacZ*] using plasmids pKHT103, pCF658 and pCF1328, respectively.

Construction of strains CF1298 [PAO1 ∆*dacB*∆*mltG attB::*P*_ampC_-lacZ att*Tn7::*P_lac_::empty*], CF1300 [PAO1 ∆*dacB*∆*mltG attB::*P*_ampC_-lacZ att*Tn7::*P_lac_::mltG*] and CF1302 [PAO1 ∆*dacB*∆*mltG attB::*P*_ampC_-lacZ att*Tn7::*P_lac_::mltG E217Q*] with P*_lac_*::empty, P*_lac_*::*mltG* and P*_lac_*::*mltG E217Q* integrated at the Tn7 locus were performed as above in strain CF1420 [PAO1∆*dacB*∆*mltG* *attB::*P*_ampC_-lacZ*] using plasmids pKHT103, pCF658 and pCF1328, respectively.

Strains CF1631 [PAO1 (empty)], CF1632 [PAO1 (P_ara_-*sltB1*-FLAG)] and CF1634 [PAO1 (P_ara_-*sltB1 E135A*-FLAG)] were obtained by electroporation of plasmids pJN105 (7), pCF1009 and pCF1010 in PAO1 [WT], respectively.

Strains CF1726 [∆*ampC* (empty)], CF1727 [∆*ampC* (P_ara_-*sltB1*-FLAG)] and CF1728 [∆*ampC* (P_ara_-*sltB1 E135A*-FLAG)] were obtained by electroporation of plasmids pJN105 (7), pCF1009 and pCF1010 in CF612 [∆*ampC*] (2), respectively.

Strains CF1731 [∆*ampR* (empty)], CF1732 [∆*ampR* (P_ara_-*sltB1*-FLAG)] and CF1733 [∆*ampR* (P_ara_-*sltB1 E135A*-FLAG)] were obtained by electroporation of plasmids pJN105 (7), pCF1009 and pCF1010 in CF550 [∆*ampR*] (2), respectively.

Strains CF1738 [∆*mltG* (empty)], CF1739 [∆*mltG* (P_ara_-*sltB1*-FLAG)] and CF1740 [∆*mltG* (P_ara_-*sltB1 E135A*-FLAG)] were obtained by electroporation of plasmids pJN105 (7), pCF1009 and pCF1010 in CF1410 [∆*mltG*], respectively.

Strains CF1638 [∆*sltB1* (empty)], CF1639 [∆*sltB1* (P_ara_-*sltB1*-FLAG)] and CF1641 [∆*sltB1* (P_ara_-*sltB1 E135A*-FLAG)] were obtained by electroporation of plasmids pJN105 (7), pCF1009 and pCF1010 in CF1105 [∆*sltB1*] (2), respectively.

**References for supplemental materials:**

1. Stover CK, Pham XQ, Erwin AL, Mizoguchi SD, Warrener P, Hickey MJ, Brinkman FS, Hufnagle WO, Kowalik DJ, Lagrou M, Garber RL, Goltry L, Tolentino E, Westbrock-Wadman S, Yuan Y, Brody LL, Coulter SN, Folger KR, Kas A, Larbig K, Lim R, Smith K, Spencer D, Wong GK, Wu Z, Paulsen IT, Reizer J, Saier MH, Hancock RE, Lory S, Olson MV. 2000. Complete genome sequence of Pseudomonas aeruginosa PAO1, an opportunistic pathogen. Nature 406:959–964.

2. Fumeaux C, Bernhardt TG. 2017. Identification of MupP as a New Peptidoglycan Recycling Factor and Antibiotic Resistance Determinant in Pseudomonas aeruginosa. mBio 8.

3. Simon R, Priefer U, Pühler A. 1983. A Broad Host Range Mobilization System for In Vivo Genetic Engineering: Transposon Mutagenesis in Gram Negative Bacteria. Bio/Technology 1:784–791.

4. Rietsch A, Vallet-Gely I, Dove SL, Mekalanos JJ. 2005. ExsE, a secreted regulator of type III secretion genes in Pseudomonas aeruginosa. Proc Natl Acad Sci U S A 102:8006–8011.

5. Hoang TT, Karkhoff-Schweizer RR, Kutchma AJ, Schweizer HP. 1998. A broad-host-range Flp-FRT recombination system for site-specific excision of chromosomally-located DNA sequences: application for isolation of unmarked Pseudomonas aeruginosa mutants. Gene 212:77–86.

6. Choi K-H, Gaynor JB, White KG, Lopez C, Bosio CM, Karkhoff-Schweizer RR, Schweizer HP. 2005. A Tn7-based broad-range bacterial cloning and expression system. Nat Methods 2:443–448.

7. Newman JR, Fuqua C. 1999. Broad-host-range expression vectors that carry the L-arabinose-inducible Escherichia coli araBAD promoter and the araC regulator. Gene 227:197–203.

8. Vieira J, Messing J. 1991. New pUC-derived cloning vectors with different selectable markers and DNA replication origins. Gene 100:189–194.

9. Caille O, Zincke D, Merighi M, Balasubramanian D, Kumari H, Kong KF, Silva-Herzog E, Narasimhan G, Schneper L, Lory S, Mathee K. 2014. Structural and functional characterization of Pseudomonas aeruginosa global regulator AmpR. Journal of Bacteriology 196:3890–3902.

10. Gibson DG, Young L, Chuang R-Y, Venter JC, Hutchison CA 3rd, Smith HO. 2009. Enzymatic assembly of DNA molecules up to several hundred kilobases. Nat Methods 6:343–345.

11. Choi K-H, Schweizer HP. 2006. mini-Tn7 insertion in bacteria with single attTn7 sites: example Pseudomonas aeruginosa. Nat Protoc 1:153–161.

12. Cavallari JF, Lamers RP, Scheurwater EM, Matos AL, Burrows LL. 2013. Changes to its peptidoglycan-remodeling enzyme repertoire modulate β-lactam resistance in Pseudomonas aeruginosa. Antimicrobial Agents and Chemotherapy 57:3078–3084.
